# Cyclipostins and Cyclophostin analogs as promising compounds in the fight against tuberculosis

**DOI:** 10.1038/s41598-017-11843-4

**Published:** 2017-09-18

**Authors:** Phuong Chi Nguyen, Vincent Delorme, Anaïs Bénarouche, Benjamin P. Martin, Rishi Paudel, Giri R. Gnawali, Abdeldjalil Madani, Rémy Puppo, Valérie Landry, Laurent Kremer, Priscille Brodin, Christopher D. Spilling, Jean-François Cavalier, Stéphane Canaan

**Affiliations:** 1 Aix-Marseille Univ, CNRS, EIPL, IMM FR3479 Marseille, France; 2 Department of Chemistry and Biochemistry, University of Missouri–St. Louis, One University Boulevard, St. Louis, Missouri 63121 United States; 3 Aix Marseille Univ, CNRS, Institut de Microbiologie de la Méditerranée FR3479, Plate-forme Marseille Protéomique (MaP), Marseille, France; 4 INSERM U1019 CNRS-UMR 8204, Institut Pasteur de Lille, Université de Lille, 1 rue du Professeur Calmette, Lille, France; 5 Institut de Recherche en Infectiologie de Montpellier (IRIM), CNRS, UMR 9004, Université de Montpellier, Montpellier, France; 6 IRIM, INSERM, 34293 Montpellier, France; 7 Present Address: Tuberculosis Research Laboratory, Institut Pasteur Korea, Seongnam-si, Gyeonggi-do 13488 Republic of Korea

## Abstract

A new class of Cyclophostin and Cyclipostins (**CyC**) analogs have been investigated against *Mycobacterium tuberculosis* H37Rv (*M. tb*) grown either in broth medium or inside macrophages. Our compounds displayed a diversity of action by acting either on extracellular *M. tb* bacterial growth only, or both intracellularly on infected macrophages as well as extracellularly on bacterial growth with very low toxicity towards host macrophages. Among the eight potential **CyCs** identified, **CyC**_**17**_ exhibited the best extracellular antitubercular activity (MIC_50_ = 500 nM). This compound was selected and further used in a competitive labelling/enrichment assay against the activity-based probe Desthiobiotin-FP in order to identify its putative target(s). This approach, combined with mass spectrometry, identified 23 potential candidates, most of them being serine or cysteine enzymes involved in *M. tb* lipid metabolism and/or in cell wall biosynthesis. Among them, Ag85A, CaeA and HsaD, have previously been reported as essential for *in vitro* growth of *M. tb* and/or survival and persistence in macrophages. Overall, our findings support the assumption that **CyC**_**17**_ may thus represent a novel class of multi-target inhibitor leading to the arrest of *M. tb* growth through a cumulative inhibition of a large number of Ser- and Cys-containing enzymes participating in important physiological processes.

## Introduction

*Mycobacterium tuberculosis* (*M. tb*) the causative agent of tuberculosis (TB) has become the number one global public health emergency worldwide. With 10.4 million new cases and 1.8 million deaths caused by *M. tb*, as reported by WHO in 2016^1^, TB is now the deadliest infectious disease around the world and remains a great challenge, especially in sub Saharan Africa, Russia and Eastern Europe. The emergence of multiple drug-resistant (MDR), extensively drug-resistant (XDR) and totally drug-resistant (TDR)^2,3^ strains over the years and the controversial results of the Gates-backed TB vaccine (MV85A)^4^ highlight the pressing need for novel therapeutic approaches^5,6^.

The key feature in the success of *M. tb* as a pathogen is its ability to evade host immunity and to establish a chronic and persistent infection^7^. Several unusual characteristics contribute to this success, the first one being its unique lipid-rich cell wall^8^. Indeed, the mycobacterial waxy coat, essential for bacterial viability and pathogenicity, possesses unique features. The complex architecture and impermeability of the cell wall are responsible for the inherent resistance of *M. tb* to many antibiotics^9^. Most current available drugs including first-line drugs such as isoniazid and ethambutol inhibit cell wall biosynthetic enzymes^5^. The same comment remains true for new antituberculosis/antibiotics currently evaluated in clinical phase II or III trials, comprising either repurposed drug or new analogues of known anti-mycobacterial drugs^6,10^. *A posteriori*, such target-specificity may not address sufficiently nor efficiently the global spreading of the disease. Following this point of view, in 2013, Zumla *et al*. stated that “*there is growing awareness of the need for drugs that can kill M. tuberculosis in its different physiological states*”^10^.

Another important issue resides in the fact that current treatments consist in a quadritherapy for 2 months, which has to be extended with a 4- to 7-months bitherapy to prevent latent TB infections (*i.e*., persisting bacilli) from turning into active TB disease^5^. The inherent difficulty to be compliant to such long treatments is in part responsible for the emergence of resistant strains and represents a new challenge to achieve control of the disease. In this context, continuous efforts for developing innovative chemotherapeutic approaches to treat TB are needed.

**Figure 1:**
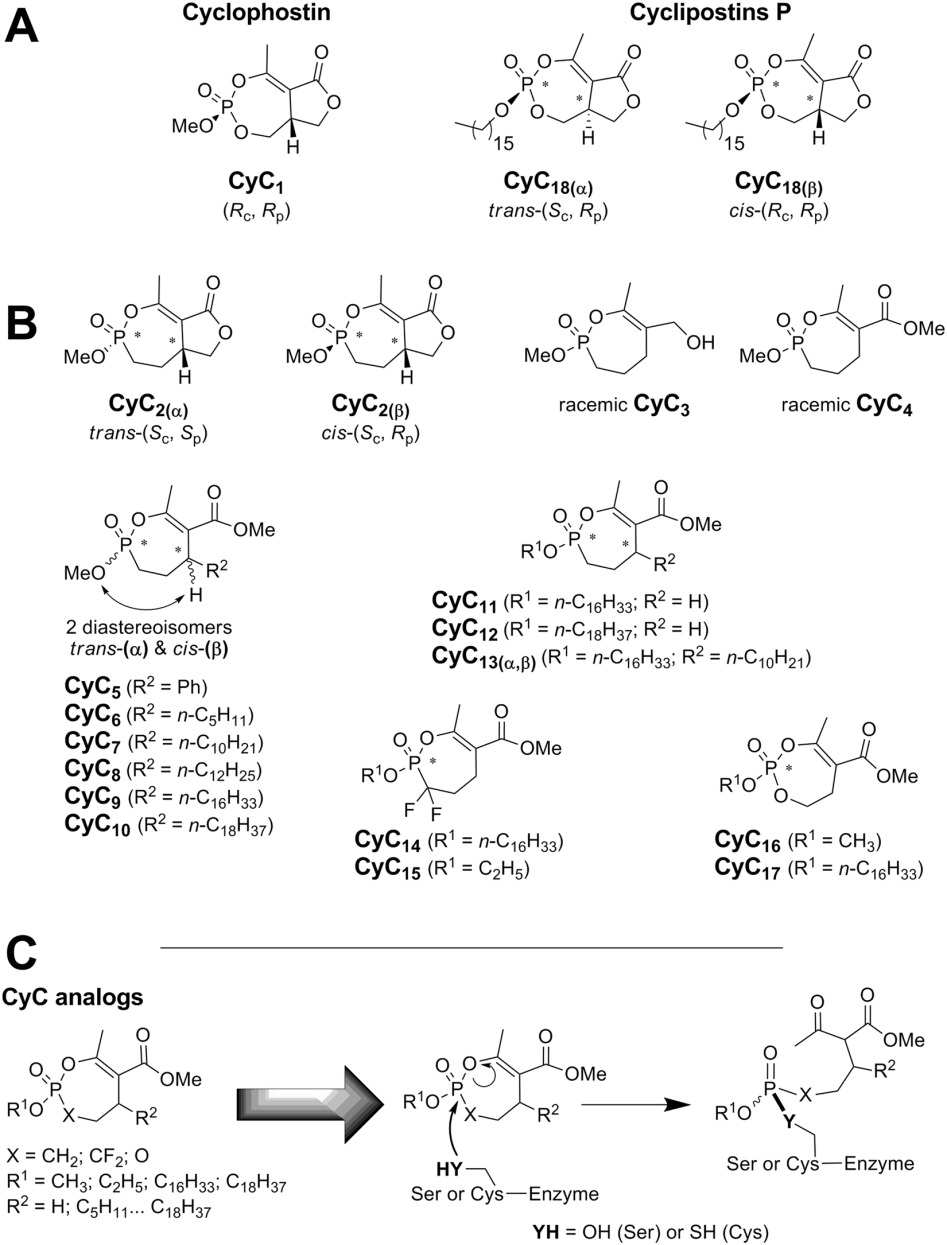
Chemical structure of CyC compounds. Structure of (**A**) natural Cyclophostin (**CyC**_**1**_), Cyclipostins P (**CyC**_**18(β)**_) and its *trans* diastereoisomer (**CyC**_**18(α)**_); as well as (**B**) the related enolphosphorus analogues: Cyclophostin phosphonate analogs (**CyC**_**2**_); monocyclic enolphosphorus analogs to either Cyclophostin (**CyC**_**3-10;15-16**_) or Cyclipostins (**CyC**_**11-14;17**_). **CyC**_**5-10**_ and **CyC**_**13**_ were best described by the relationship between the OMe on phosphorus and the H-substituent on the C-5 carbon atom as being either in a *trans* (α-isomer) or *cis* (β-isomer) relationship. (**C**) Mode of action of **CyC** analogs. All **CyC** compounds are able to form a covalent adduct with the nucleophilic serine or cysteine catalytic residues present at the active site of α/β-hydrolase enzymes family.

Analogues of natural Cyclophostin (**CyC**_**1**_) and Cyclipostins (*e.g*., natural Cyclipostins P: **CyC**_**18(β)**_) (**CyC** compounds - Fig. 1) appear as prime candidates to be tested against *M. tb*. These natural compounds, isolated from fermentation of *Streptomyces sp*.^11,12^, have been reported to inhibit growth of various mycobacteria such as *Mycobacterium smegmatis*, *Mycobacterium phlei*, *Nocardia abcessus* as well as *Corynebacterium diphteriae* with similar minimum inhibitory concentrations (MIC) than those of rifampicin and penicillin G^13^. From a chemical point of view, Cyclipostins family members possess a bicyclic enol-organophosphorus core structure similar to that of Cyclophostin, but are phosphate esters of long chain lipophilic alcohols (Fig. 1A).

These natural compounds were also shown to be potent inhibitors of either acetylcholinesterase (*i.e*., Cyclophostin)^11,14^ or human hormone-sensitive lipase (*i.e*., Cyclipostins)^12,15^.

We have previously reported the total synthesis of natural Cyclophostin (**CyC**_**1**_) and Cyclipostins P (**CyC**_**18**_)^15^ and their respective biological activity against purified lipolytic enzymes. Similar studies were conducted with their phosphate (**CyC**_**16-17**_) and phosphonate (**CyC**_**2-15**_) analogs^14,16–19^ (Fig. 1B). These studies led to the conclusion that, upon nucleophilic attack by a catalytic serine or cysteine residue, a covalent bond is formed between the enol-phosphorous atom and the catalytic residue as depicted in Fig. 1C ^16,17^.

Moreover, modulation of the lipophilicity by varying the nature and chain length of the alkyl group either at the C-5 carbon atom (*i.e*., R^2^ group – Fig. 1) or at the phosphorous center (*i.e*., R^1^ group – Fig. 1), strongly impacted the inhibitory efficiency of the **CyC** and could be exploited further to either decrease or increase the affinity of one inhibitor to target a specific enzyme over others^17^. Consequently, these **CyC** analogs have not only proved to be powerful mycobacterial enzyme inhibitors; but above all they had lost their inhibitory activity on acetylcholinesterase and human hormone-sensitive lipase, which correspond to the mammalian enzymes initially targeted by the natural **CyC** compounds^17–19^. This promises a great potential for these cyclic enolphosph(on)ate analogs of Cyclophostin (and the Cyclipostins) as a new class of selective serine/cysteine enzyme inhibitors in mycobacteria. The selectivity of the **CyC** derivatives to inhibit the mycobacterial but not the human enzymes, is therefore highly valuable and prompted us to consider these compounds as potential antitubercular agents.

Herein, each **CyC** molecule has been tested against *M. tb* for ***i***) its capacity to inhibit *in vitro* growth; ***ii***) its antitubercular activity on *M. tb*-infected macrophages, and ***iii***) its eventual cytotoxicity towards macrophages. Unexpectedly, whereas few analogs were found to inhibit *M.tb* growth *in vitro* and in macrophages similarly to isoniazid, they all showed absence of toxicity in mammalian cells. Importantly, potential targets of **CyC**_**17**_, the most potent inhibitor, were identified *via* an activity-based protein profiling (ABPP) approach, and further validated by the constructions of overexpressing mycobacterial strains.

## Results

### Synthesis of CyC analogs

To further complete the already available library of 26 **CyC** compounds (*i.e*., **CyC**_**1-12, 14-18**_)^14,15,17–19^ and to significantly improve the lipophilicity, **CyC**_**13**_ was synthesized by introducing simultaneously a C_16_-side alkyl chain (*i.e*., R^1^ group) and a C_10_-side alkyl chain at the C-5 carbon atom (*i.e*., R^2^ group), leading to an hybrid compound between **CyC**_**7**_ and **CyC**_**11**_ (Fig. 1).

### Antitubercular activity and toxicity of the CyC compounds

**Figure 2:**
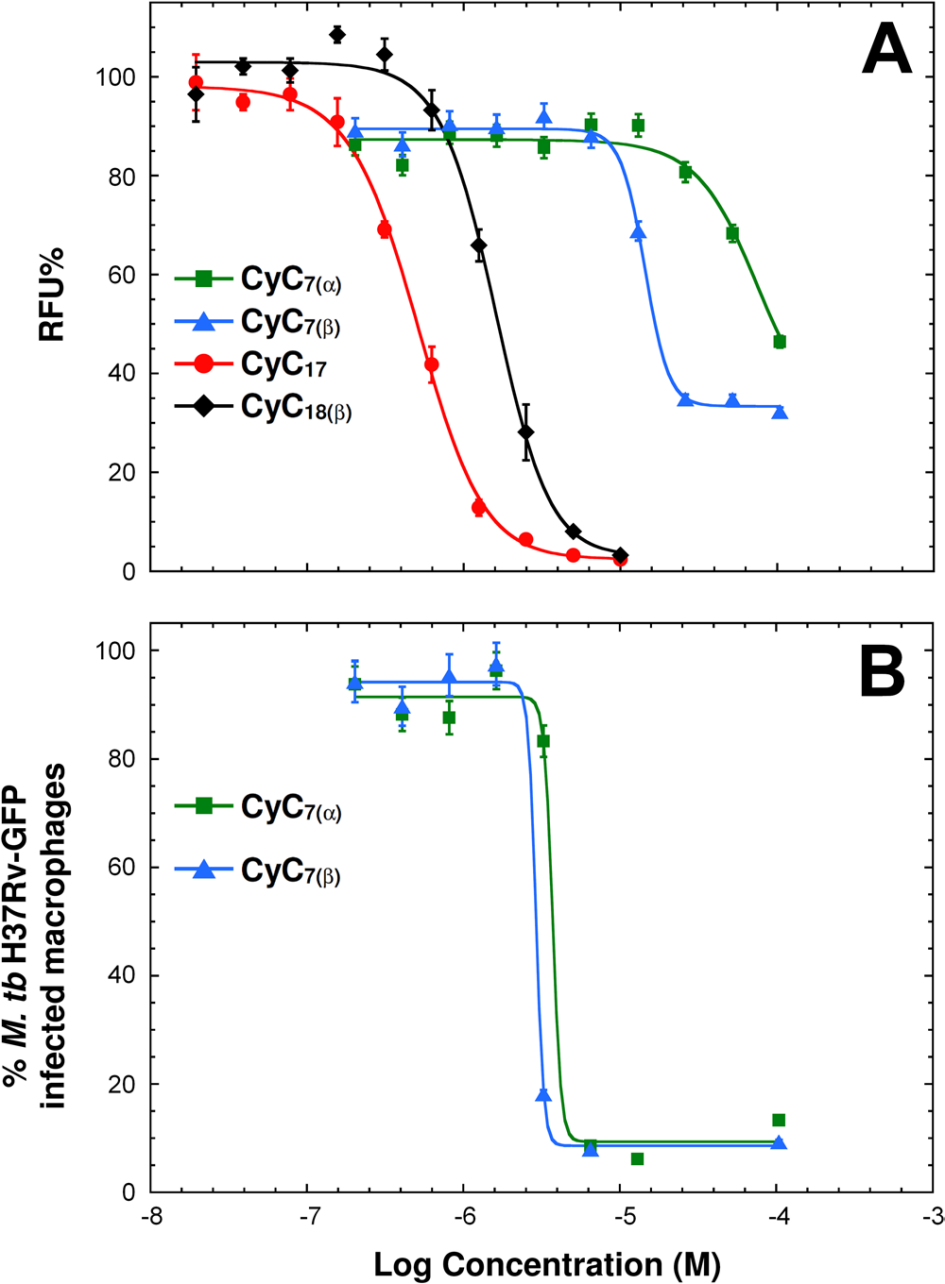
*In vitro* and *ex vivo* dose-response activity of the CyC analogs against *M. tb* H37Rv. (**A**) Activity of **CyC**_**7(α)**_, **CyC**_**7(β)**_, **CyC**_**17**_, and **CyC**_**18(β)**_ against GFP-expressing *M. tb* replicating in broth medium, expressed as normalized relative fluorescence units (RFU%). (**B**) Activity of **CyC**_**7(α)**_ and **CyC**_**7(β)**_ against *M. tb* replicating inside Raw264.7 macrophages. Results are expressed as the percentage of infected macrophages after 5 days post-infection. For each concentration, data are means ± SD of at least two independent assays performed in duplicate. The MIC_50_ of **CyC**_**17**_, **CyC**_**18(β)**_, **CyC**_**7(β)**_ and **CyC**_**7(α)**_ replicating in culture broth medium were 0.5 μM, 1.7 µM, 16.6 µM and 92.6 μM, respectively. The MIC_50_ of **CyC**_**7(α)**_ and **CyC**_**7(β)**_ replicating inside macrophages were 4.5 μM and 3.1 μM, respectively. Values are means ± SD of three independent assays performed in triplicate (CV% < 5%).

The set of 27 **CyC** analogs were first evaluated for their antitubercular activity in a high-content screening assay based on H37Rv-GFP reporter strain^20^. *In vitro* growth of *M. tb* H37Rv-GFP was monitored by directly measuring fluorescence emission after 5 days at 37 °C in the presence of increasing drug concentrations. Intracellular growth of *M. tb* H37Rv-GFP was also assessed following a 5-day exposure of infected Raw264.7 murine macrophages to the different compounds. In the latter case, the percent of infected cells and the number of living host cells allowed to simultaneously determine the MIC_50_ (concentration leading to 50% growth inhibition) and the CC_50_ (concentration leading to 50% host cell toxicity) as reported earlier^20,21^. Among the 27 analogs, eight potential candidates exhibited very promising antitubercular activities (Table 1 and Fig. 2). Interestingly, **CyC**_**7(β)**_ and **CyC**_**8(α)**_ exhibited moderate (16–40 µM) and good (3–4 µM) activity against extracellular and intramacrophagic *M. tb*, respectively. In contrast, **CyC**_**6(β)**_, **CyC**_**7(α)**_ and **CyC**_**8(β)**_ appeared to be active only on infected macrophages; whereas **CyC**_**17**_ and Cyclipostins P, *i.e*. **CyC**_**18(α)**_ and **CyC**_**18(β)**_, impaired selectively *M. tb* growth in culture broth medium with MIC_50_ up to the nanomolar range (MIC_50_ ≅ 500 nM for **CyC**_**17**_). More particularly, both **(α)** and **(β)** isomers of **CyC**_**7**_ as well as **CyC**_**8(α)**_ were found to exhibit similar or higher MIC_50_ values towards intramacrophagic bacilli than the first line antibiotics used as references (Table 1).

**Table 1:** Antibacterial activities of the most active **CyC** analogs^a^.

Compounds		Extracellular growth	Intracellular macrophage growth^b^	
		MIC_50_ (µM)	MIC_50_ (µM)	CC_50_ (µM)
Isoniazid (INH)^c^		1.2	1.2	>150
Ethionamide (ETO)^c^		6.0	6.0	120
Rifampicin (RIF)^c^		0.01	2.9	24
**CyC**_**6(β)**_		*No effect*	12.6	>100
**CyC**_**7(α)**_		92.6	**4.5**	>100
**CyC**_**7(β)**_		16.6	**3.1**	>100
**CyC**_**8(α)**_		40.4	**4.0**	>100
**CyC**_**8(β)**_		>100	**11.7**	>20
**CyC**_**17**_		**0.50**	*No effect*	>100
**CyC**_**18(α)**_		24.4	*No effect*	>100
**CyC**_**18(β)**_		**1.7**	*No effect*	>100

^a^Experiments were performed as described in Materials and Methods. MIC_50_: compound minimal concentration leading to 50% growth inhibition. CC_50_: compound concentration leading to 50% host cell toxicity. The best MIC_50_ obtained are highlighted in bold. Values are means of three independent assays performed in triplicate (CV% < 5%). ^b^Raw264.7 macrophages were infected by *M. tb* H37Rv-GFP at a MOI of 2. ^c^Data from^20^.

Beside antibacterial activity, significantly, all the latter inhibitors displayed very low toxicity towards host macrophages, with cytotoxic concentration (CC_50_) >100 μM, similarly to isoniazid (CC_50_ > 150 µM) and ethionamide (CC_50_ ≥ 120 µM), two potent antitubercular agents.

Regarding the newly synthesized analog, varying at the same time both R^1^ and R^2^ alkyl side chains did not yield any significant antibacterial activity of the resulting **CyC**_**13**_ compared to the parent **CyC**_**7**_ compound.

### Targets identification - Activity-based protein profiling (ABPP) approach

One of the major hurdles in drug development resides in the identification of the target(s) of small molecules selected from whole cell screens. The abovementioned results with the **CyC** analogs, acting either against extracellular and/or intracellular mycobacteria, suggest the possibility of several mechanism of action. This would also imply that multiple enzymes may be targeted by these compounds, resulting in the inhibition of bacteria growth.

This prompted us to apply an Activity-based protein profiling (ABPP) approach^22^ for targets identification. The so-called activity-based probes (ABPs), following labelling and enrichment procedures, allows to isolate selective sets of low-copy-number enzymes in complex proteomic mixtures through the chemical recognition of a specific catalytic mechanism without interference from the more represented proteins^22^. ABPs typically contain (*i*) a reactive group which forms a covalent and irreversible adduct with the target; (*ii*) a linker region that allows to control the specificity of the probe; and (*iii*) a tag for visualization (fluorescent tag)^23^ and/or enrichment and isolation^24^ of the covalently labelled proteins.

Considering the structure and mode of action of the 8 selected **CyC** analogs on catalytic serine or cysteine active residues (Fig. 1C), chemically relevant fluorophosphonate (FP) ABPs, bearing either a fluorophore (*i.e*., rhodamine for TAMRA-FP) or a biotin (*i.e*., Desthiobiotin-FP) reporter tag, were selected (Figure S3)^25,26^. Due to their mechanism of action leading to irreversible enzyme inhibition (Figure S3), such ABPs have been exploited to screen for reversible and irreversible inhibitors of drug targets^27–30^.

**Figure 3:**
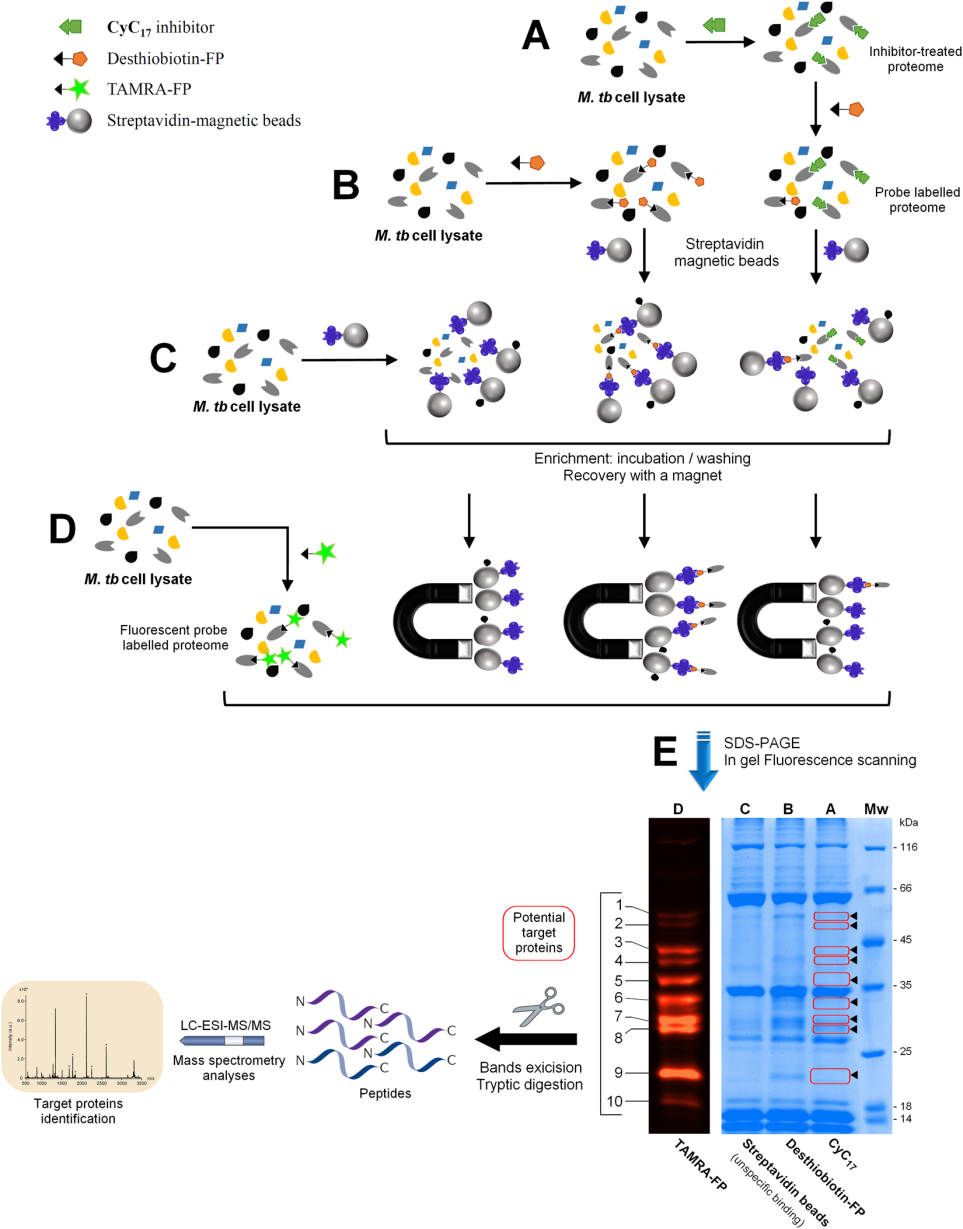
Activity based protein profiling (ABPP) workflow for the identification of the proteins covalently bound to CyC_17_ inhibitor. Cell lysates of *M. tb* mc^2^ 6230 were either (**A**) pre-treated with CyC_17_ prior to incubation with Desthiobiotin-FP probe or (**B**) incubated with Desthiobiotin-FP alone. Both samples were further treated with streptavidin-magnetic beads for the capture and enrichment of labelled proteins. (**C**) Uncompetitive binding assay using streptavidin-magnetic beads on cell lysate. (**D**) Detection of all potential serine/cysteine enzymes in total cell lysate using fluorescent TAMRA-FP probe. (**E**) Equal amounts of proteins obtained in A to D were separated by SDS-PAGE and visualized by Coomassie staining (*right* panel – lanes A–C) or in-gel fluorescence (*left* panel - lane D: TAMRA detection). Enzymes whose labelling is impeded because of the presence of CyC_17_ in the active-site are circled in red and shown by arrowheads. The corresponding bands were excised form the gel and subjected to triptic digestion and tandem mass spectrometry analysis. The SDS gel presented in panel E is representative of three independent ABPP experiments.

Here, compound **CyC**_**17**_, exhibiting the best antitubercular activity on extracellular *M. tb* growth, was selected for competitive probe labelling/enrichment assay by Desthiobiotin-FP using crude lysates of *M. tb* mc^2^6230 (Fig. 3A–C). In parallel, TAMRA-FP labelling (Fig. 3D) was used to reveal most, if not all, serine/cysteine enzymes present in the lysate, presumably reacting with **CyC**_**17**_.

Ten distinct bands unraveled by TAMRA-FP labelling were clearly visible in the fluorescence readout (Fig. 3E – lane **D**) and could also be detected in Coomassie blue staining after capture/enrichment of total lysate by Desthiobiotin-FP (Fig. 3E – lane **B**). In contrast, pre-treatment with **CyC**_**17**_ (Fig. 3A) resulted in a decrease in intensity of bands 1, 2 and 8; or disapearance of bands 3-7 and 9. (Fig. 3E – lane **A**). Indeed, the enzymes previously inactivated by **CyC**_**17**_ inhibitor will thus be unable to further react with the FP-ABP. Proteins corresponding to bands 1-9 were then excised from the gel, digested with trypsin and the resulting peptides were analyzed by liquid chromatography-tandem mass spectrometry (LC-MS/MS) for subsequent protein identification. To overcome the potential overlap of proteins, the proteins that were also present at the same position in the control experiment (*i.e*., lane **D**: DMSO alone for unspecific binding to streptavidin-magnetic beads) have not been taken into account, therefore leading to 23 distinct protein candidates (Table 2). Each protein was assigned on the basis of the numbers of unique peptides, the total number of identified peptide spectra matched, and the corresponding molecular weight (Table S1).

**Table 2:** CyC_17_ target proteins identified in *M. tb* mc^2^6230 lysate by LC-ESI-MS/MS^a^.

Band	Protein name	Rv number	kDa	Essentiality	Function	Functional Category^b^	ref.
1	Amidase **AmiC**	Rv2888c	50.9		Amidase	IM/R	—
	Amidase **AmiB2**	Rv1263	49.1		Amidase	IM/R	—
	D-3-phosphoglycerate dehydrogenase (PGDH) **SerA1**	Rv2996c	54.5	*in vitro*	Methyltransferase	IM/R	^32^
	Carboxylesterase A **CaeA**	Rv2224c	55.9	Macrophage and *in vitro* growth	Lipase/esterase	CW/CP	^49,51^
2	Penicillin-binding protein	Rv1730c	55.8		β-lactamase	CW/CP	^30^
	Serine hydroxymethyltransferase 1 (SHM1) **glyA1**	Rv1093	46.2	*in vitro*	Methyltransferase	IM/R	^33^
3	L-lactate dehydrogenase **LldD2**	Rv1872c	45.3		Dehydrogenase	IM/R	^31^
	Esterase **LipM**	Rv2284	46.7		Lipase/esterase	IM/R	—
	Lipase **LipE**	Rv3775	45.2		Lipase/esterase	IM/R	—
4	hypothetical protein **LH57_07490**	Rv1367c	43.7		β-lactamase	CW/CP	—
	Lipase/esterase **LipN**	Rv2970c	40.1		Lipase/esterase	IM/R	^36^
	Alcohol dehydrogenase **AdhB**	Rv0761c	39.7		Dehydrogenase	IM/R	—
5	Lipase **LipH**	Rv1399c	34.0		Lipase/esterase	IM/R	^37^
	Secreted antigen 85-A FbpA **Ag85A**	Rv3804c	35.7	*in vitro*	Lipase/esterase	LM	^45,46^
	Secreted antigen 85-C FbpC **Ag85C**	Rv0129c	36.7		Lipase/esterase	LM	^45,47^
6	Putative hydrolase	Rv0045c	32.1		Lipase/esterase	LM	^43^
	Mycolic acid synthase **UmaA**	Rv0469	33.1		Methyltransferase	LM	^34^
	Hydrolase **hsaD**	Rv3569c	32.1	Macrophages and growth on cholesterol	Hydrolase	IM/R	^53,54^
7	Monoglyceride lipase	Rv0183	30.2		Lipase/esterase	IM/R	^39,40^
	Thioesterase **tesA**	Rv2928	29.1	*in vitro*	Lipase/esterase	LM	^48^
8	Lipase **LipV**	Rv3203	27.9		Lipase/esterase	IM/R	^38^
	Putative non-heme bromoperoxidase **BpoC**	Rv0554	28.4		Hydrolase	V/D/A	^35^
9	Cutinase **Culp1**	Rv1984c	21.8		Lipase/esterase	CW/CP	^41, 42^

^a^The 9 excised bands from the typical SDS-PAGE gel depicted in Fig. 3E were digested by trypsin followed by LC-MS/MS analysis. Only proteins not present in control incubations (DMSO alone for unspecific binding to streptavidin-magnetic beads) were included in this list. Positive hits were selected as described in Materials and Methods. ^b^IM/R: Intermediary metabolism/respiration; CW/CP: cell wall/cell processes; LM: Lipid metabolism; V/D/A: Virulence, detoxification, adaptation.

As expected from previous ABPP studies on *M. tb* proteome^29,30^, the FP probe recognized a wide range of serine and cysteine enzymes. Here, the identified enzyme candidates ranged in their functional category from intermediary metabolism/respiration (13 proteins), lipid metabolism (5 proteins), cell wall/cell processes (4 proteins), and virulence/detoxification/adaptation (1 protein) (Table 2).

Enzymes involved in metabolic processes included the alcohol dehydrogenase AdhB (Rv0761c) thought to catalyze the reversible oxidation of ethanol to acetaldehyde with the concomitant reduction of NAD; the putative L-lactate dehydrogenase LidD2 (Rv1872c)^31^; the methyltransferase SerA1 (Rv2996c) involved in the L-serine biosynthetic process^32^; glyA1 (Rv1093) annotated as a serine hydroxymethyltransferase with possible role in serine to glycine conversion^33^; and UmaA (Rv0469) a S-adenosyl-L-methionine-dependent methyltransferase capable of catalyzing the conversion of phospholipid-linked oleic acid to essential tuberculostearic acid^34^, a major constituent of mycobacterial membrane phospholipids. Little is known about the catalytic reactions of these enzymes. However, our results, in line with previous findings using fluorophosphonate ABPs^29,30^, suggest the presence of at least one nucleophilic (catalytic?) serine or cysteine residue involved in the formation of a covalent adduct with **CyC** inhibitors.

The remaining 18 enzymes belong to the serine/cysteine hydrolase family proteins. Among them, a few hydrolases were identified: two putative β-lactamases Rv1730c (currently annotated as a possible penicillin-binding protein) and Rv1367c, both possibly involved in cell wall biosynthesis; two amidases AmiC (Rv2888c) and AmiB2 (Rv1263); and BpoC a possible peroxidase (Rv0554)^35^ recently proposed as being a functional serine hydrolase^30^. Five members of the lipase family Lip (LipE, LipH, LipM, LipN^36^, and LipV) were detected; a number significantly lower than the 13 active *M. tb* Lip enzymes reported using Desthiobiotin-FP^29^ or the 8 lipases using an alkyne-PEG-FP probe^30^. Among the five captured Lip proteins, LipH (Rv1399c)^37^ and LipV (Rv3203)^38^ had been functionally characterized previously. LipH is known to hydrolyze short-chain ester and may participate in the detoxification pathway of the intracellular lipid metabolism while LipV posseses a broad range substrate specificity and is also active at low pH, suggesting a role in *M. tb*’s adaptation to acidic conditions into the phagosome. Beside members of the Lip family, six additional enzymes with lipolytic activity were isolated: Rv0183, a monoacylglycerol lipase that degrades host-cell lipids^39,40^; Cfp21 (Rv1984c), a cutinase-like protein preferentially active against medium-chain carboxylic esters and monoacylglycerols^41,42^; as well as the esterase Rv0045c^43^ proposed to participate in lipid hydrolysis.

Five additional lipolytic enzymes appeared as highly promising target candidates of **CyC**_**17**_: the antigen 85 complex Ag85A (Rv3804c) and Ag85C (Rv0129c), the thioesterase TesA (Rv2928), the carboxylesterase CaeA (Rv2224c) and the hydrolase HsaD (Rv3569c); the latter two proteins being annotated as essential enzymes^44^.

Ag85A and Ag85C express both a mycolyl transferase activity. They catalyze the transfer of mycolic acids from trehalose monomycolate (TMM) to produce trehalose dimycolate (TDM) and are also responsible for the covalent attachment of mycolic acids to arabinogalactan^45,46^. Moreover, inhibition of Ag85C was found to block TDM synthesis and to disrupt the integrity of the cell envelope^47^. Similarly, TesA has been found to be required for the synthesis of both phenolic glycolipids and phthiocerol dimycocerosate (PDIM). Inactivation of *TesA* in *M. marinum* was correlated with an important decrease in virulence and increase susceptibility to drugs^48^. CaeA (also named Hip1 for hydrolase important for pathogenesis 1) is a cell wall-associated carboxylesterase involved in cell wall biosynthesis and/or integrity^49^. CaeA was also found to play important roles in virulence^49^, multidrug-resistance^50^ and innate immunity^51^. The absence of CaeA enhanced host innate immune responses and compromised the intracellular survival of *M. tb* in macrophages^52^. The hydrolase HsaD was first described as participating in cholesterol catabolism^53^ and then found to be essential for intramacrophage survival of *M. tb* ^51^. HsaD has recently been proposed as a novel therapeutic target and awaits further developments^54^.

### Functional Validation: Overexpression of Target Proteins Leads to Reduced Susceptibility to CyC_17_

Genes encoding Ag85A, Ag85C, Rv0183, LipH, TesA and HsaD were cloned and overexpressed in *M. tb* (Table S2). These six genes were choosen as representative candidates for their involvement in mycobacterial lipid metabolism and/or for their importance during the bacteria life cycle. Overexpression of each individual protein was confirmed by Western blotting as compared to the WT strain (Figure S4).

To examine whether these six overexpression strains were affected on their susceptibility to **CyC**_**17**_, MIC_50_ of **CyC**_**17**_ were determined for each strain. Whereas overexpression of Ag85A, Ag85C, Rv0183 or TesA did not show significant changes in MIC_50_ compared to the vector control and parental strain (WT) (Table 3), overexpression of either LipH or HsaD was associated with increased resistance levels to **CyC**_**17**_. Compared to WT strain (MIC_50_ = 0.55 ± 0.023 μM), overexpression of LipH caused a slight increase in the MIC_50_ value of around 1.3-fold (0.72 ± 0.020 μM; *p-value* < 0.001), while overexpression of HsaD led to a significant 2.2-fold increase in MIC_50_ value (1.20 ± 0.026 μM; *p-value* < 0.001).

**Table 3:** MIC_50_ of CyC_17_ against *M. tb* mc^2^6230 overexpression strains.

Overexpression strains	MIC_50_ (µM)	MIC_50_ ratio mutant/WT
*M. tb* mc^2^6230 WT	0.55 ± 0.023^‡,†^	1.00
*M. tb* mc^2^6230-empty vector	0.52 ± 0.010	0.95
*M. tb* mc^2^6230-Ag85A	0.55 ± 0.014	1.00
*M. tb* mc^2^6230-Ag85C	0.54 ± 0.009	0.98
*M. tb* mc^2^6230-Rv0183	0.44 ± 0.013	0.80
*M. tb* mc^2^6230-LipH	**0.72 ± **0.020^*,‡^	**1.31**
*M. tb* mc^2^6230-TesA	0.52 ± 0.012	0.95
*M. tb* mc^2^6230-HsaD	**1.20 ± **0.026^*,†^	**2.18**

^a^Experiments were performed as described in Materials and Methods. MIC_50_: compound minimal concentration leading to 50% growth inhibition. Values are mean of at least two independent assays performed in triplicate (CV% < 5%). MIC_50_ values with a commun symbol (*,^‡^,^†^) are significantly different (*p-value* < 0.001; ANOVA followed by Fisher’s test).

### Modelling the potential CyC_17_ binding site in HsaD

The increased MIC_50_ value of the strain overexpressing HsaD prompted us to explore the potential interactions occurring at the enzyme’s active site following **CyC**_**17**_ binding. *In silico* molecular docking experiments were conducted, as described previously^17^ using the recently reported crystal structures of HsaD bound to three different inhibitors^54^: 3,5-dichloro-4-hydroxybenzoic acid (PDB id: 5JZS), 3,5-dichloro-4-hydroxybenzenesulphonic acid (PDB id: 5JZ9) and 3,5-dichloro-benzenesulfonamide (PDB id: 5JZB).

**Figure 4:**
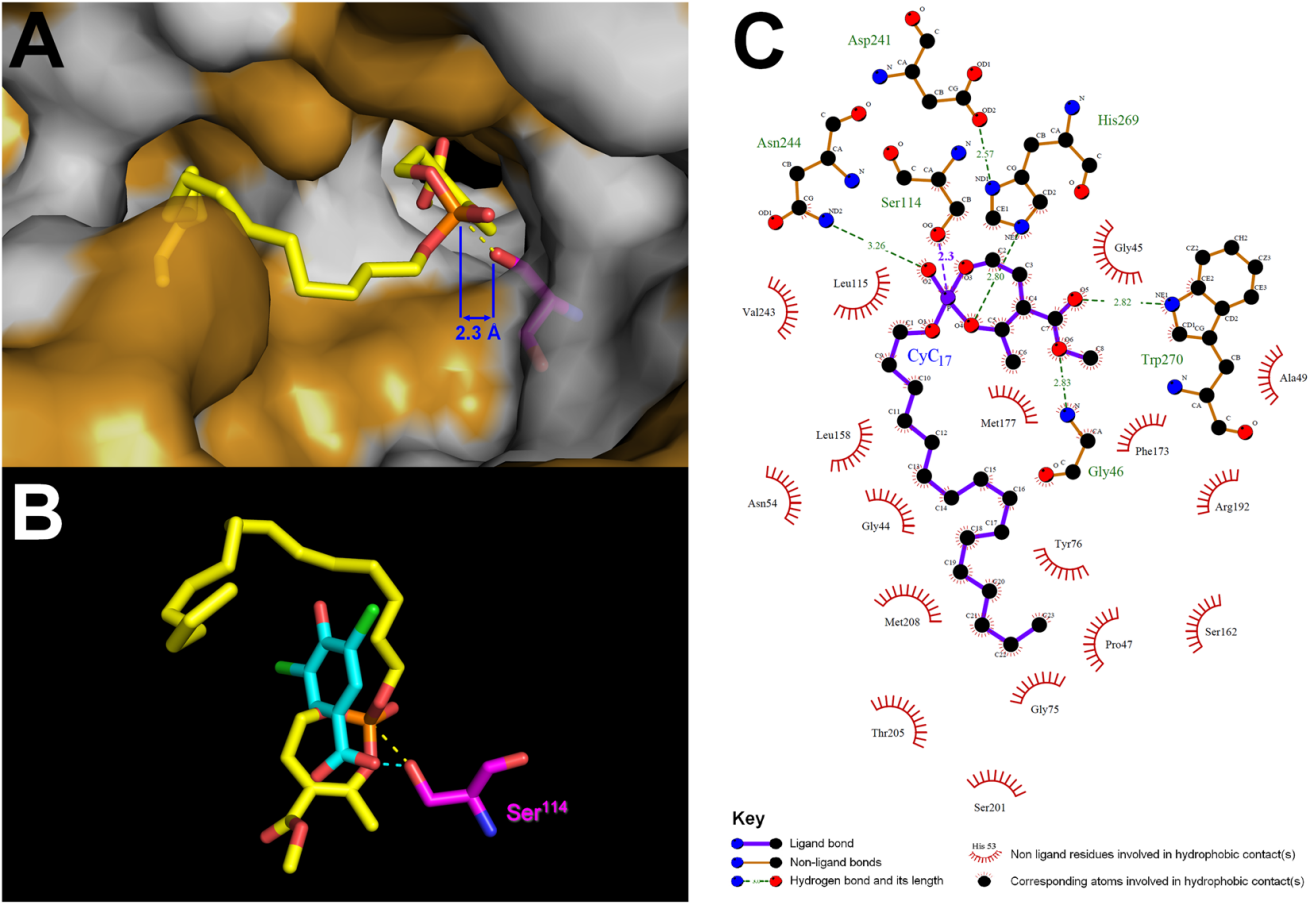
*In silico* molecular docking experiments. (**A**) *In silico* molecular docking of **CyC**_**17**_ into the crystallographic structure of HsaD in a van der Waals surface representation. Hydrophobic residues (alanine, leucine, isoleucine, valine, tryptophan, tyrosine, phenylalanine, proline and methionine) are highlighted in white. (**B**) Superimposition of the top-scoring docking position of **CyC**_**17**_ (yellow) with the crystal structure of 3,5-dichloro-4-hydroxybenzoic acid (cyan) found to bind in the vicinity of the catalytic Ser^114^ of HsaD. Each inhibitor is in stick representation with the following atom color-code: oxygen, red; phosphorus, orange; carbon, yellow or cyan; chloride, green. The catalytic Serine residue is colored in magenta. Structures were drawn with PyMOL Molecular Graphics System (Version 1.4, Schrödinger, LLC) using the PDB file 5JZS^54^. (**C**) Ligplot + analyses results: 2D representation of schematic ligand-protein interactions of **CyC**_**17**_ in HsaD active site showing both hydrogen-bonds and hydrophobic interactions.

The best scoring position obtained (*i.e*., lowest energy complex) indicated that the reactive seven-membered monocyclic enolphosphorus ring adopted a productive orientation (Fig. 4). The reactive phosphorous atom of the inhibitor was indeed found in a position facilitating the occurrence of a reaction with the catalytic Ser^114^ (d[Ser-Oγ/P = O] distance <2.5 Å) and thus the formation of a covalent bond. It is also noteworthy that a high level of concordance was observed between this favorable docked conformation of **CyC**_**17**_ and the structure of the 3,5-dichloro-4-hydroxybenzoic acid found to bind in the vicinity of the HsaD active site (Fig. 4B).

The docked **CyC**_**17**_-HsaD complex was then subjected to interactions analysis using Ligplot + v.1.4^55^ (Fig. 4C). The Ligplot + diagram schematically depicts the hydrogen bonds and hydrophobic interactions between the ligand (*i.e*., **CyC**_**17**_) and the active site residues Ser^114^-Asp^241^-His^269^ of the protein during the binding process. The Ligplot + analysis clearly shows that the reactive phosphorous atom is stabilized by H-bonding with Asn^244^ and His^269^ residues (Fig. 4C). Moreover, 17 hydrophobic contacts could be detected and appear critical to stabilize the inhibitor inside the HsaD active site (Fig. 4A and C). The C_16_-side alkyl chain perfectly accommodate the hydrophobic pocket opposite to the catalytic Ser^114^ residue, and interacts with Gly^44^, Pro^47^, Asn^54^, Gly^75^, Tyr^76^, Leu^115^, Leu^158^, Ser^162^, Ser^201^, Thr^205^, Met^n^ and Val^n^ residues. The seven-membered enolphosphate ring, located in a distinct pocket, is stabilized by two H-bonding with Gly^n^ and Trp^270^, and interacts with Gly^n^, Ala^49^, Phe^173^, Met^177^ and Arg^192^ residues.

From these findings, **CyC**_**17**_ may thus bind to HsaD in a very similar orientation and with clear overlapping areas of interaction than the previously reported HsaD-bound inhibitors (*i.e*., 3,5-dichloro-4-hydroxybenzoic acid, 3,5-dichloro-4-hydroxybenzenesulphonic acid and 3,5-dichloro-benzenesulfonamide)^54^. Specifically, all residues involved in H-bonding and hydrophobic contacts in each of the above mentioned complex structures are also present in stabilizing **CyC**_**17**_, therefore reinforcing the accuracy of our model. Taken together, this network of interactions presumably allows the formation of a stable and productive binding mode, and might provide a clear picture of the inhibition of HsaD by **CyC**_**17**_.

## Discussion

Drug discovery developments to generate new lead compounds along with their corresponding targets and mode of action represent a major need in the “*fight*” against TB. Herein, we have evaluated the anti-tubercular activities of a set of 27 **CyC** analogs (Fig. 1), that were initially designed to inhibit mycobacterial lipolytic enzymes^17^. It is now well established that lipolytic enzymes, involved in the host-pathogen cross-talk, play critical roles in the physiopathology of the disease and participate in the entry into a non-replicating dormant state within host granulomas and/or in dormancy escape, leading to reactivation of the disease and virulence^56–58^. Indeed, *M. tb* triggers the formation of lipid bodies (LB) inside infected macrophages, providing the cells a foamy appearance^59^. In foamy macrophages (FM), bacilli accumulate lipids within intracytoplasmic lipid inclusions (ILI)^7^, which allow the bacteria to persist in a non-replicating state. To persist inside FM, *M. tb* hydrolyzes host lipids triacylglycerols (TAG) from LB into fatty acids that are reprocessed as lipid reserves within ILI^7,59^. During the reactivation phase, these ILIs are hydrolyzed by *M. tb* and used to fuel the replication of mycobacteria during their exit from the hypoxic non-replicating state^60^. Therefore, finding ways to inhibit the activity of such mycobacterial lipolytic enzymes may pave the way for discovering new modalities for TB treatment.

Some known lipase inhibitors such as the oxadiazolone M*m*PPOX compound^61^, Orlistat^61,62^, or more recently the human lysosomal acid lipase inhibitor Lalistat^28^, have already been described to block *M. tb* growth with MICs ranging from 25-50 μM. Despite these moderate inhibition activities, a strong synergistic effect on *in vitro M. tb* growth was reached for the combined application of both latter inhibitors with vancomycin, resulting in a MIC drop of 16-fold for Orlistat (MIC~6 µM)^62^ and 4-fold for Lalistat (MIC~6–12 µM)^28^.

In our study, among the 27 tested **CyC** analogs, eight showed moderate (16–40 µM), potent (3–4 µM) or very good (0.5 µM) activity as judged by their MIC_50_ values (Table 1). Unexpectedly, this set of 8 analogs can be divided into two classes. **CyC**_**6(α)**_, **CyC**_**7(α,β)**_ and **CyC**_**8(α,β)**_ showed a clear preference against intracellularly-replicating mycobacteria. This supposes that the intracellular mode of action of this class of inhibitors differs from that of those acting exclusively on extracellularly-replicating bacilli. It can therefore be hypothesized that vulnerability of the corresponding target(s) of these inhibitors becomes more apparent and critical during the intracellular lifestyle of *M. tb*. A specific response of the macrophage induced by the action of these compounds and leading to bacterial death cannot however be excluded. In contrast, **CyC**_**17**_ and **CyC**_**18(α,β)**_ showed high activity exclusively on extracellular bacteria, a property already observed previously for 1,2,4-Oxadiazole EthR Inhibitors^21^ and was correlated to limited bioavailability and to the hydrophilicity of these compounds. From a structure-activity relationships (SAR) perspective, some trends have emerged with respect to the effects of the **CyC** analogs tested. Regarding the natural Cyclophostin (**CyC**_**1**_) and its phosphonate derivatives **CyC**_**2-10;15-16**_, it is noteworthy that identified bioactive compounds **CyC**_**7(α,β)**_ and **CyC**_**8(α,β)**_ bearing medium C_10_- and C_12_-side alkyl chains, respectively, are corresponding to the most potent and also “*less*” selective inhibitors of various bacterial enzymes, as compared to the other **CyC** analogs which were found to exhibit a greater selectivity towards pure recombinant mycobacterial lipase over human counterparts^17^. With Cyclipostin P, the potent antibacterial activity of **CyC**_**18(α,β)**_ is in good agreement with the *in vitro* growth inhibition reported earlier on various mycobacteria^13^. Moreover, the fact that only the monocyclic enolphosphate **CyC**_**17**_ displays antituberculous activity when compared to the non-active enolphosphonate derivatives **CyC**_**11**_, **CyC**_**12**_ and **CyC**_**14**_, emphasizes the specific need for a phosphate moiety in such heptacyclic analogs to exhibit bactericidal activity against *M. tb* growth *in vitro*.

Another interesting finding of this work is related to the fact that among the 8 most active **CyC** tested (Table 1), only the phosphonates **CyC**_**6-8**_ were found active against *M. tb* in macrophages. It is indeed well known that phosphates are susceptible to hydrolysis by alkaline phosphatases, whereas the corresponding phosphonates are stable to unwanted hydrolysis, which increases their lifetimes *in vivo* ^63^. Consequently, the apparent difference between the extracellular and the intracellular modes of action may simply rely on chemical properties of the phosphate *vs*. phosphonate chemical groups. Based on the aforementioned results, one can assume that **CyC** inhibitors would profoundly alter the outcome of the infection by impairing mycobacterial growth within host cells. In addition, they may also affect the entry of bacilli into the persistence phase and/or interfere with reactivation of dormant bacilli in macrophages. In contrast to other lipase inhibitors such as Orlistat or Lalistat, a major improvement of the **CyC** molecules resides in the fact that they may react exclusively with bacterial enzymes^17–19^ with no cytotoxic effects towards host macrophages.

To gain access to the mechanisms of action, an ABPP approach was successfully applied allowing the identification of mycobacterial enzymes impaired by the inhibitors during mycobacterial growth. Selective labelling and enrichment of captured enzymes using appropriate fluorophosphonate probes in combination with **CyC**_**17**_ resulted in the identification of 23 potential target enzymes (Fig. 3 and Table 2). As anticipated, all identified proteins were serine or cysteine enzymes, thus validating the approach. All these 23 enzymes have already been identified from ABPP experiments on *M. tb* lysates with non-specific fluorophosphonate probes^29,30^. It is also noteworthy that the later three essential enzymes (*i.e*., Ag85A, CaeA and HsaD) were also captured from *M. bovis* BCG lysates using an Orlistat-alkyne analog and click chemistry for targets enrichment^27^; they were not detected, however, when a Lalistat-like probe was directly incubated with *M. tb* cells prior to lysis and chemical proteomics. Such finding is in agreement with a complementary target profile exerted by each lipase inhibitor given their respective physico-chemical properties. Since the MIC of these two lipase inhibitors towards *M. tb* growth (around 25-50 μM) was however 50- to 100-times higher than that of **CyC**_**17**_ (0.5 µM), it is thus tempting to speculate that the shared preference for a specific set of enzymes is responsible for the high growth inhibitory potency of our **CyC** monocyclic enolphosphate.

To validate the targets of **CyC**_**17**_, genes encoding the identified targets (Ag85A, Ag85C, Rv0183, LipH, HsaD or TesA) were overexpressed in *M. tb* mc^2^6230. Whereas overexpression of LipH or HsaD led to slight, but statistically significant increased resistance levels, thereby suggesting that these two lipolytic enzymes could be effective drug targets; overexpression of Rv0183, Ag85A, Ag85C or TesA did not change the susceptibility/resistance profile to **CyC**_**17**_ (Table 3). This further strengthens the hypothesis that this inhibitor, and presumably the other **CyC** analogs, represent multi-target agents. Consequently, individual overexpression of single potential target enzyme is unlikely to generate high resistance level. Accordingly, by blocking at the same time the activities of various lipolytic enzymes, such as LipH, Rv0183 and HsaD, on the one hand; and those of TesA, Ag85A and Ag85C on the other hand **CyC**_**17**_ would strongly interfere with the acquisition and consumption of host cell-derived lipids by the mycobacteria, and also destabilize the cell envelope assembly. In such conditions, such a large spectrum of inhibitory effects exerted by our **CyC** analogs cannot be considered as a weakness if only *M. tb* is impacted, and on the contrary can open new avenues for the treatment of TB. Above all, this work led to the identification of very promising anti-TB candidates that should be able to act against bacteria in various physiological stages, thus allowing a faster sterilization.

## Conclusion

A priority for new drug-development to efficiently treat TB must be focused on the discovery of novel therapeutic targets and approaches. In this work, we evaluated the antitubercular activities of a series of Cyclipostins and Cyclophostin (**CyC**) analogs both *in vitro*, and *ex vivo* in infected macrophages. This led to the selection of a set of promising **CyC** candidates that are devoid to cytotoxic properties towards host cells. By targeting multiple enzymes either involved in lipid metabolism and/or in cell wall biosynthesis, these compounds are emerging as a novel class of multi-target anti-TB candidates which should open up new chemotherapeutic opportunities in the fight against TB. By blocking extracellular and/or intracellular *M. tb* growth, we anticipate these compounds could prevent the entry of *M. tb* in the persistence phase and/or reactivation of dormant bacilli residing within the granuloma and the foamy macrophages. To our knowledge, there is no other family of compounds able to target and impair replicating bacteria as well as intracellular bacteria. The dual activity of the **CyC** inhibitors is of major importance as it may affect the different stages of the infection process. Because lipid storage in bacteria is thought to drive the infection process, **CyC** inhibitors can also be viewed as attractive candidates to further dissect the fate of the bacteria in the context of infected foamy macrophages.

## Materials and Methods

### Synthesis of Cyclophostin and Cyclipostins molecules

The synthesis of natural Cyclophostin **CyC**_**1**_^15^, their phosphonate analogs **CyC**_**2(α)**_ and **CyC**_**2(β)**_^14^, the monocyclic enolphosphonates **CyC**_**3-4**_^16^ and the *trans*-(α) and *cis*-(β) diastereoisomers **CyC**_**5-10**_^17^; as well as the *trans*-(α) and *cis*-(β) Cyclipostin P **CyC**_**18**_^15^ and the corresponding monocyclic phosphonate **CyC**_**11-12**_^17^, difluorophosphonate **CyC**_**14-15**_ and phosphate **CyC**_**16-17**_^18,19^ analogs were obtained at 98% purity as described previously. Stock solutions (10 mM) in which the **CyC** compounds were found to be completely soluble in dimethyl sulfoxide (DMSO), were prepared prior to extracellular and intracellular drug susceptibility testing.

The new lipophilic enolphosphonate **CyC**_**13**_ was prepared *via* a transesterification reaction from racemic **CyC**_**7**_ using established techniques already reported for **CyC**_**16-17**_ synthesis^19^, giving desired compound as a mixture of diastereoisomers. Briefly a solution of **CyC**_**7**_ (27 mg, 0.072 mmol) in 1,4-dioxane (360 µL) was added to a flask containing tetrabutylammonium iodide (TBAI; 2.7 mg, 0.0073 mmol, 0.1 equiv.) followed by hexadecyl bromide (220 µL, 0.72 mmol, 10 equiv.). The flask was placed in an oil bath preheated to 105 °C. After 4.5 hours, the solution was cooled and concentrated *in vacuo*. The residue was purified by column chromatography (SiO_2_, 8% EtOAc in hexane) to give the oily product (38.4 mg, 91% yields) as a mixture of *trans*-(α) and *cis*-(β) diastereoisomers. The two isomers were further separated by preparative reversed phase HPLC (C18 column, 100% MeOH) as follows. Preparative HPLC Specifications and Conditions. Manual preparative injector: Rheodyne 1700 (3725i-119) with 20 mL loop; Solvent A – MeOH; Solvent B – H_2_O; Varian ProStar Model 210 pumps equipped with 25 mL/min Rainin/Gilson type pump heads. Kromasil 100-10C18-2025 column; 10 µm particle diameter; 250 mm × 20 mm i.d. Spectra-Physics Spectra 100 UV detector with prep cell. LKB 2211 Superac fraction collector. 100% MeOH at a flow rate of 10 mL/min.

The HPLC data were supported by careful analysis of the^1^H, ^13^C, and particularly the ^31^P NMR spectra, and high resolution mass spectrometry (Figures S1–S2).

Fast eluting isomer **CyC**_**13(β)**_ (15.8 mg). HPLC RT 38 min; IR (neat, NaCl) 2293, 2853 1718, 1651 cm^−1^; ^1^H NMR (300 MHz, CDCl_3_) *δ* 4.19 (1 H, m), 4.06 (1 H, m), 3.75 (3 H, s), 2.90 (1 H, m), 2.17 (3 H, d, *J*_HP_ = 2.1 Hz), 2.15–1.85 (4 H, m), 1.75–1.45 (4 H, m), 1.30 (42 H, m), 0.88 (6 H, overlapping t, *J*_HH_ = 6.3 Hz); ^13^C NMR (75.4 MHz, CDCl_3_) *δ* 169.3, 155.1 (d, *J*_CP_ = 9.0 Hz), 123.1 (d, *J*_CP_ = 4.6 Hz), 66.5 (d, *J*_CP_ = 6.5 Hz), 51.9, 37.5, 32.1 (d, *J*_CP_ = 1.5 Hz), 31.3, 30.7 (d, *J*_CP_ = 5.5 Hz), 29.9–29.7 (multiple overlapping peaks), 29.6 (d, *J*_CP_ = 2.0 Hz), 29.4, 27.9, 25.7, 25.6 (d, *J*_CP_ = 7.6 Hz), 23.9, 22.8, 22.1, 21.3 (d, *J*_CP_ = 2.5 Hz), 14.3; ^31^P NMR (121.4 MHz, CDCl_3_) *δ* 22.1 ppm; HRMS (FAB, NBA, MH^+^) calcd for C_34_H_66_O_5_P: 585.4648, found 585.4664. Slow eluting isomer **CyC**_**13(α)**_ (17.8 mg). HPLC RT 48 min; IR (neat, NaCl) 2923, 2853, 1718, 1652 cm^−1^; ^1^H NMR (300 MHz, CDCl_3_) *δ* 4.13 (2 H, m), 3.73 (3 H, s), 2.98 (1 H, m), 2.22 (3 H, d, *J*_HP_ = 1.6 Hz), 2.15–1.85 (4 H, m), 1.75–1.45 (4 H, m), 1.30 (42 H, m), 0.88 (6 H, overlapping t, *J*_HH_ = 6.9 Hz); ^13^C NMR (75.4 MHz, CDCl_3_) *δ* 169.2 (d, *J*_CP_ = 1.7 Hz), 156.1 (d, *J*_CP_ = 7.3 Hz), 123.2 (d, *J*_CP_ = 5.1 Hz), 66.1 (d, *J*_CP_ = 7.0 Hz),, 52.0, 37.4, 32.1 (d, *J*_CP_ = 1.1 Hz), 30.9, 30.6 (d, *J*_CP_ = 6.1 Hz), 29.9–29.7 (multiple overlapping peaks), 29.5 (d, *J*_CP_ = 2.1 Hz), 29.3, 27.8, 25.7, 25.1 (d, *J*_CP_ = 6.7 Hz), 23.3, 22.9, 21.6, 21.5, 14.3; ^31^P NMR (121.4 MHz, CDCl_3_) *δ* 24.9 ppm; HRMS (FAB, NBA, MH^+^) calcd for C_34_H_66_O_5_P: 585.4648, found 585.4634.

### Bacterial strains and growth conditions

For intra and extracellular assays, *M. tb* H37Rv expressing GFP^20^ was grown for 14 days in 7H9 medium (Difco) supplemented with 10% oleic acid-albumin-dextrose-catalase (OADC, BD Difco), 0.5% glycerol, 0.05% Tween 80 and 50 µg/mL hygromycin B (Euromedex). For target identification, the experiments were conducted using *M. tb* mc^2^6230 (H37Rv *ΔRD1 ΔpanCD)* a derivative of H37Rv which contains a deletion of the RD1 region and *panCD*, resulting in a pan(−) phenotype^64^ *. M. tb* mc^2^6230 was grown in 7H9 medium supplemented with 10% OADC (BD Difco), 0.5% glycerol, 0.05% Tween 80 and 24 µg/mL D-panthothenate (Sigma-Aldrich). Cultures were kept at 37 °C without shaking.

### Intracellular assay

The growth of *M. tb* H37Rv-GFP strain in macrophages was monitored by automated fluorescence confocal microscope (Opera, Perkin-Elmer) as already described^20^. Briefly, bacteria were washed twice with PBS and resuspended in RPMI 1640 medium (Invitrogen) supplemented with 10% heat-inactivated fetal bovine serum (FBS, Invitrogen). Murine (Raw264.7) macrophages were infected at a multiplicity of infection (MOI) of 2:1 and incubated 2 hours at 37 °C in RPMI 1640 medium containing 10% FBS. Cells were then washed, treated with 50 µg/mL amikacin (Euromedex) for 1 hour at 37 °C to kill all extra-cellular bacteria, washed again and finally seeded in 384-well plates (5 × 10^5^ cells/mL), containing 2-fold dilutions of compounds in DMSO. The final volume of DMSO was kept under 0.3%. Plates were incubated for 5 days at 37 °C, 5% CO_2_. Infected cells were stained for 30 min using Syto60 dye (Invitrogen) at a final concentration of 5 µM before reading using fluorescence confocal microscope (20X water objective; GFP: λ_ex_ 488 nm, λ_em_ 520 nm; Syto60: λ_ex_ 640 nm, λ_em_ 690 nm). Sigmoidal dose-response curves were fitted using Prism software (sigmoidal dose-response, variable slope model). The concentration required to inhibit 50% of *M. tb* intracellular growth (MIC_50_) was determined using ten-point dose-response curves as an average of the MIC_50_ of all parameters, the ratio of infected cells and the bacterial area per infected cell.

### Extracellular assay

A 14 days old culture of *M. tb* H37Rv-GFP was washed twice with PBS and resuspended in 7H9 medium containing 10% OADC, 0.5% glycerol, 0.05% Tween 80 and 50 µg/mL hygromycin B. Bacteria were seeded in 384 well plates (7 × 10^5^ bacteria/mL) containing 2-fold dilutions of the compounds in DMSO. The final volume of DMSO was kept under 0.3%. Plates were incubated at 37 °C, 5% CO_2_ for 5 days. Bacterial fluorescence levels (RFU) were recorded using a fluorescent microplate reader (Victor × 3, Perkin-Elmer). The MIC_50_ of all tested compounds were determined using ten-point dose-response curves.

### Activity-Based Protein Profiling (ABPP) approach for target enzymes identification

#### Preparation of lysates for ABPP experiments

From 1 L of culture at the logarithmically growth stage (OD_600_~1), *M. tb* mc^2^6230 cells were harvested by centrifugation at 4,000 g for 15 min. Pellets were washed 3 times with PBS containing 0.05% Tween 80. The cell pellets were resuspended in PBS buffer at a 1:1 (w/v) ratio. The bacterial cells were then mixed with the same volume of 0.1 mm diameter glass beads (BioSpec) and disrupted during 4 min of violent shaking using Mini-Beadbeater-96 (BioSpec). The lysate was then centrifuged at 4 °C and at 12,500 g for 15 min to remove the cell debris and unbroken cells. Supernatants were adjusted to a concentration of 2 mg/mL of total proteins, snap frozen in liquid nitrogen and stored at −80 °C until further use.

#### In-gel detection of total M. tb potential target enzymes using TAMRA-FP probe

*M. tb* mc^2^6230 lysates (50 µL–100 µg total proteins) were incubated with 2 μM ActivX TAMRA-FP probe (ThermoFisher Scientific) or DMSO (unlabelled control) for 90 min at room temperature and in absence of light. The reaction was stopped by adding 5X Laemmli reducing sample buffer and boiling at 95 °C for 5 min. The labelled proteins were further analyzed by SDS-PAGE electrophoresis (12% Bis-Tris gel) followed by fluorescent gel scanning (TAMRA: λ_ex_ 557 nm, λ_em_ 583 nm) and detection using the Cy^®^3 filter of a ChemiDoc MP Imager (Bio-Rad). Alternatively, the gel was stained with Coomassie blue R250 staining solution and was destained with solution of 10% ethanol and 30% acetic acid.

#### Identification of M. tb potential target enzymes of CyC_17_ using Desthiobiotin-FP probe

*M. tb* mc^2^6230 lysates (500 µL–1 mg total proteins) were incubated with 2 µM ActivX Desthiobiotin-FP probe (ThermoFisher Scientific) or DMSO (unlabelled control) for 90 min at 37 °C. For inhibitor studies, lysates were pre-incubated with 580 µM **CyC**_**17**_ at 37 °C for 90 min prior to Desthiobiotin-FP treatment. The reaction was next stopped by adding 0.3 g of urea (10 M final concentration) to denature proteins completely. Unreacted probes were removed using Zeba Spin desalting column (7 K MWCO, ThermoFisher Scientific) and labelled proteins were further captured by 200 µg Nanolink streptavidin magnetic beads 0.8 µm (Solulink), according to the manufacturer’s instructions.

First, 20 µL of a 10 mg/mL NanoLink streptavidin magnetic beads was transferred into a 1.5 mL Eppendorf tube. The Wash Buffer (50 mM Tris-HCl, 150 mM NaCl, 0.05% Tween 20, pH 8.0) was then added to bring the final volume to 250 µL and the resulting mixture was mixed gently to resuspend and wash the beads. The tube was placed on a magnetic stand for 2 min. and the supernatant was discarded. The tube was removed from the magnetic stand and the beads were washed two more times with the Wash Buffer (250 µL). Each *M. tb* mc^2^6230 treated-lysate sample was enriched for labelled proteins by transfer to the previously washed beads (around 200 µg). The lysate/beads suspensions were incubated for 1 hour at room temperature with mild shaking. The tubes were then placed on the magnetic stand for 2 min to collect the beads, and the supernatant was removed. The beads containing bound, biotinylated proteins were washed three time carefully with the Wash Buffer, as described above, and resuspended in 25 µL PBS buffer pH 7.4 containing 50 mM free biotin. The resulting solution was mixed with 5X Laemmli reducing sample buffer, and heated at 95 °C for 5 min. This step allowed the recovery of the captured labelled proteins by exchanging the initially captured desthiobiotin/streptavidin complex to the greater affinity biotin/streptavidin complex.

The released proteins were resolved by SDS-PAGE at 160 V for 1 hour. The gel was stained with Coomassie blue R250 staining solution and was destained with solution of 10% ethanol; 30% acetic acid. To check for unspecific binding, a DMSO-treated lysate sample was incubated only with the streptavidin-magnetic beads in absence of Desthiobiotin-FP probe treatment, and processed as described above.

### Target enzymes identification *via* mass spectrometry analyses

#### Peptide analysis by mass spectrometry

The bands of interest were first excised from gels. Classical steps of washes (100 mM ammonium bicarbonate/acetonitrile, 50:50 *v/v*) were followed by reduction (10 mM dithiothreitol for 1 h. at 56 °C), alkylation (55 mM iodoacetamide for 30 min at room temperature) and digestion by a trypsin solution (10 ng/µL, Promega) containing ProteaseMAX 0.025% (*w/v*) (Promega) in 50 mM ammonium bicarbonate overnight at 37 °C. Tryptic peptides were extracted by 0.1% TFA in water/acetonitrile (50:50 *v/v*) and dried into a speed vacuum. Mass spectrometry was performed on a Q Exactive Plus mass spectrometer (ThermoFisher Scientific, Bremen, Germany) equipped with a nanospray ion source and coupled to an Ultimate 3000 nano UPLC (Dionex, ThermoScientific, Sunnyvale, CA, USA). Dried tryptic peptides were dissolved in 2% acetonitrile/0.05% TFA in water and desalted on a C18 µ-precolumn (PepMap100, 300 µm × 5 mm, 5 µm, 100 Å, Dionex) before elution onto a C18 column (Acclaim PepMap, RSLC, 75 µm × 150 mm, 2 µm, 100 Å, Dionex). Peptides were eluted with a linear gradient from 6 to 40% of mobile phase B (20% water, 80% acetonitrile/0.1% formic acid) in A (0.1% formic acid in water) for 52 min. Peptides were detected with a workflow combining full MS (350- 1900 *m/z* range at 70000 resolution)/data dependent MS/MS Top 10 (high collision dissociation, 150 –2250 *m/z* range).

#### Database searching for identification of CyC_17_ target enzymes

Mass spectra were processed using Proteome Discoverer software v. 2.1.0.81 (ThermoFisher Scientific) based on SequestHT algorithm. The following parameters were used: organism, UniProt *M. tuberculosis* H37Rv database (GI TaxID = 83332, v2016-08-20, 5535 entries); enzyme, trypsin; missed cleavages, 2; dynamic modification, Oxidation Met + 15.995 Da; static modification, Carbamidomethyl Cys + 57.021 Da; minimum length of peptides, 6 amino acids; precursor mass tolerance, 10 ppm; fragment mass tolerance, 0.02 Da. Proteins were considered as identified by at least two unique peptides passing the high confidence filter (Relaxed Target FDR:0.05 and Maximum Delta Cn: 0.05). For more details about proteins identification, *i.e*. sequence coverage and number of identified peptides see Table S1.

### Functional validation of selected target enzymes

#### Construction of M. tb mc^2^6230 strains overexpressing Ag85A, Ag85C, Rv0183, LipH, HsaD or TesA

*ag85A (Rv3804), ag85C (Rv0129c), rv0183, lipH* (*Rv1399c), hsaD (Rv3569c)* and *tesA (Rv2928)* were amplified by PCR from *M. tb* H37Rv genomic DNA. Specific primers (listed in Table S2) were used to integrate either the NdeI (for *rv0183*, *lipH*, *hsaD* and *tesA*) or the MscI (for *ag85A* and *ag85C*) restriction site at the 5′ end and BamHI at the 3′ end for all the genes. Amplicons were digested with the corresponding restriction enzymes (ThermoFisher Scientific), gel purified using Nucleospin Gel and PCR Clean-up kit (Macherey-Nagel) and cloned into proper restriction sites of pMV261 (for *ag85A, ag85C*) or pVV16 in frame with a C-Terminus 6-His tag (for *rv0183*, *lipH*, *hsaD* and *tesA*), both harbouring the *hsp60* promoter. The DNA sequences of each insert were confirmed by DNA sequencing (GATC Biotech).

#### Preparation of competent cells

*M. tb* mc^2^6230 electrocompetent cells were prepared as described previously by Goude *et al*.^65^. Briefly, 100 mL of *M. tb* mc^2^6230 cells were cultivated up to mid-log phase (*i.e*., OD_600_~0.6) and glycine was added to a final concentration of 0.2 M and incubated during 16 hours. Cells were harvested, washed four times with 10% glycerol solution at room temperature and finally resuspended in 1/100 of the original volume. 200 µL of competent cells were mixed with 1 µg of DNA and transferred to a 2 mm gap electroporation cuvette. A single pulse of 2.5 kV, 25 µF with resistance set at 600 Ω was provided. Culture media was immediately added to the mycobacterial suspension and then incubated during 24 hours at 37 °C. Bacteria were plated on 7H10 Middlebrook agar supplemented with 10% OADC and 50 µg/mL of both kanamycin and hygromycin. Plates were incubated at 37 °C during 3 weeks. Positive transformants were further grown in liquid medium up to OD 1 and the overexpression of the recombinant proteins was checked by Western blot using either the specific monoclonal antibody Mab 32/15 kindly provided by Dr. K. Huygen directed against the *M. tb* Ag85 complex^66^, specific rabbit polyclonal antibodies directed against Rv0183^39^, or HisProbe HRP conjugated (ThermoFisher Scientific) for the other proteins.

#### Resazurin microtiter assay (REMA) for drug susceptibility

Susceptibility testing was performed in 7H9 medium supplemented with 10% OADC, 0.5% glycerol, 0.05% Tween 80, 24 µg/mL D-panthothenate and kanamycin (50 µg/mL) when needed. Assays were carried out in triplicate. MICs of each *M. tb* mc^2^6230 mutant strains overexpressing Ag85A, Ag85C, Rv0183, LipH, HsaD or TesA were determined in 96-well flat-bottom Nunclon Delta Surface microplates with lid (ThermoFisher Scientific, ref. 167008) using the resazurin microtiter assay (REMA^67,68^). Briefly, log-phase bacteria (*i.e*., OD_600_ ~ 1–1.5) were diluted to a cell density of 5 × 10^6^ CFU/mL. Then 100 µL of the above inoculum was added to each well containing 100 µL 7H9 medium, serial two-fold dilutions of **CyC**_**17**_ or controls to a final volume of 200 µL (final bacterial charge of 2.5 × 10^6^ CFU/mL per well). Growth controls containing no inhibitor (*i.e*., bacteria only = *B*), inhibition controls containing 50 µg/mL isoniazid (Euromedex) and sterility controls (*i.e*., medium only = *M*) without inoculation were also included. Plates were incubated at 37 °C in a humidity chamber^69^ to prevent evaporation. After 10–14 days of incubation, 20 µL of a 0.020% (*w/v*) resazurin (Sigma-Aldrich) solution was added to each well, and the plates were incubated at 37 °C for 24 hours for color change from blue to pink or violet and for a reading of fluorescence units (FU). Fluorescence corresponding to the resazurin reduction was quantified using a Tecan Spark 10 M multimode microplate reader (Tecan Group Ltd, France) with excitation at 530 nm and emission at 590 nm. For fluorometric MIC determinations, a background subtraction was performed on all wells with a mean of *M* wells. Relative fluorescence unit was define as: RFU% = (test well FU/mean FU of *B* wells) × 100. MIC values were determined by fitting the RFU% sigmoidal dose-response curves in Kaleidagraph 4.2 software (Synergy Software). The lowest drug concentrations inhibiting 50% of growth were defined as the MIC_50_.

## Electronic supplementary material

Supporting information

Identified Proteins
