# Supplementary material for: Cyclipostins and Cyclophostin analogs as promising compounds in the fight against tuberculosis
Source: Sci Rep. 2017 Sep 18;7:11751. doi: 10.1038/s41598-017-11843-4 (PMC5603573; doi:10.1038/s41598-017-11843-4)
Supplement: Supplementary file 1 — Supporting information [file 41598_2017_11843_MOESM1_ESM.pdf]

Supporting Information

Cyclipostins and Cyclophostin analogs as promising compounds in the fight against tuberculosis

Phuong Chi Nguyen[1], Vincent Delorme[4 #], Anaïs Bénarouche[1], Benjamin P. Martin[2], Rishi Paudel[2], Giri R. Gnawali[2], Abdeldjalil Madani[1], Rémy Puppo[3], Valérie Landry[4], Laurent Kremer[5,6], Priscille Brodin[4], Christopher D. Spilling[2], Jean-François Cavalier[1*] and Stéphane Canaan[1*]

[1] Aix-Marseille Univ, CNRS, EIPL, *IMM FR3479*, Marseille, France

[2] Department of Chemistry and Biochemistry, University of Missouri−St. Louis, One University Boulevard, St. Louis, Missouri 63121, United States

[3] Aix Marseille Univ, CNRS, Institut de Microbiologie de la Méditerranée FR3479, plate-forme Marseille Protéomique (MaP), Marseille, France

[4] INSERM U1019 CNRS-UMR 8204, Institut Pasteur de Lille, Université de Lille, 1 rue du Professeur Calmette, Lille, France

[5] Institut de Recherche en Infectiologie de Montpellier (IRIM), CNRS, UMR 9004, Université de Montpellier

[6] IRIM, INSERM, 34293, Montpellier, France

[*] Correspondance: canaan@imm.cnrs.fr (S. Canaan); jfcavalier@imm.cnrs.fr (J.-F. Cavalier)

[#] *Current Address: Tuberculosis Research Laboratory, Institut Pasteur Korea, Seongnam-si, Gyeonggi-do, 13488 Republic of Korea.*

Contents

^{1}H, ^{13}C NMR and ^{31}P NMR spectra of **CyC$_{13(\alpha)}$** (**Figure S1**) S2

^{1}H, ^{13}C NMR and ^{31}P NMR spectra of **CyC$_{13(\beta)}$** (**Figure S2**) S5

Primers used in this study (**Table S2**) S8

Chemical structures of TAMRA-FP and Desthiobiotin-FP (**Figure S3**) S9

Western blot analysis of *M. tb* mc^{2}6230 overexpression strains (**Figure S4**) S10

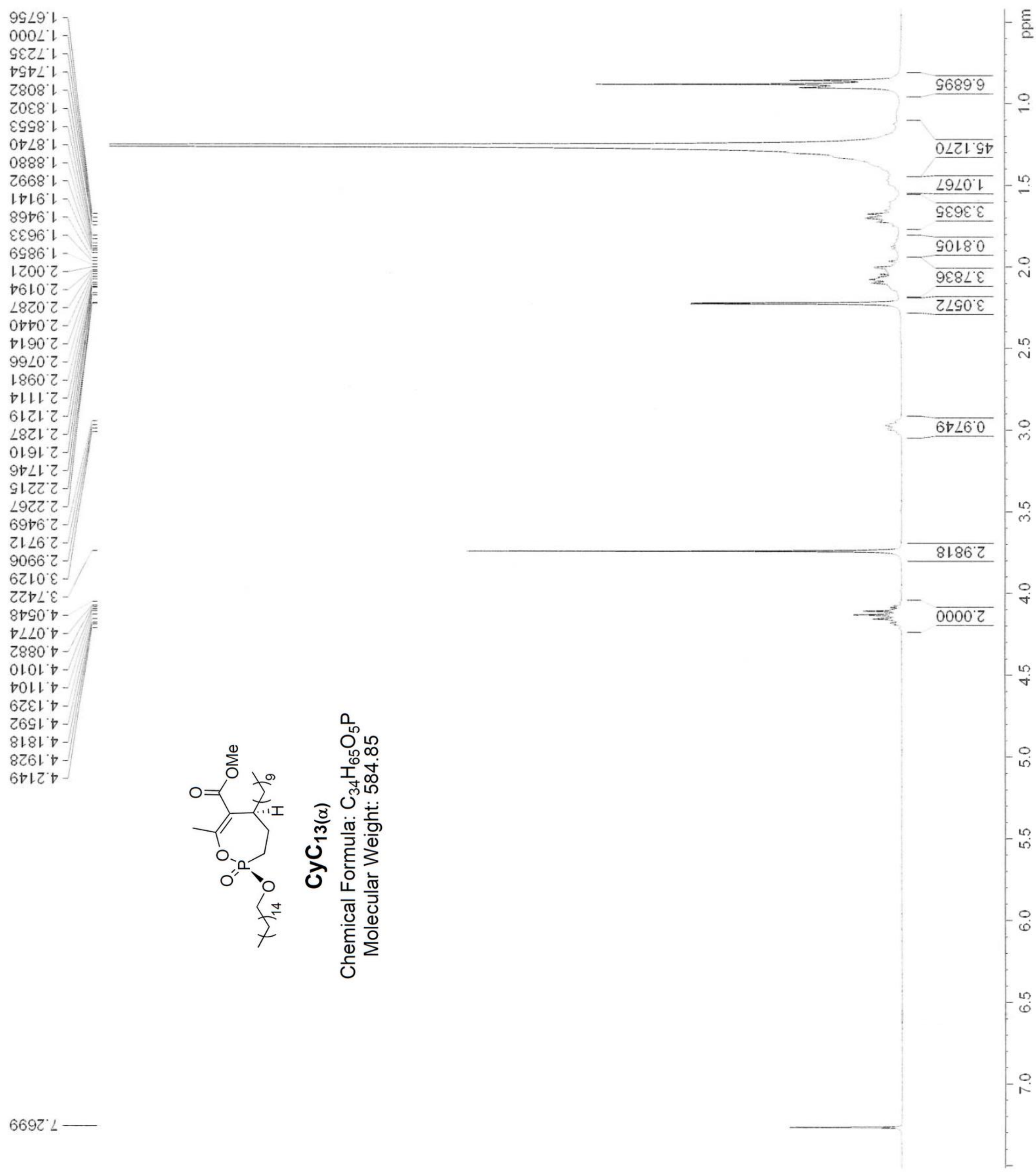

Figure S1A. ^{1}H NMR spectrum of **CyC$_{13(\alpha)}$** recorded at 300 MHz, and using $CDCl_3$ (δ = 7.27 ppm) as an internal standard of solvent.

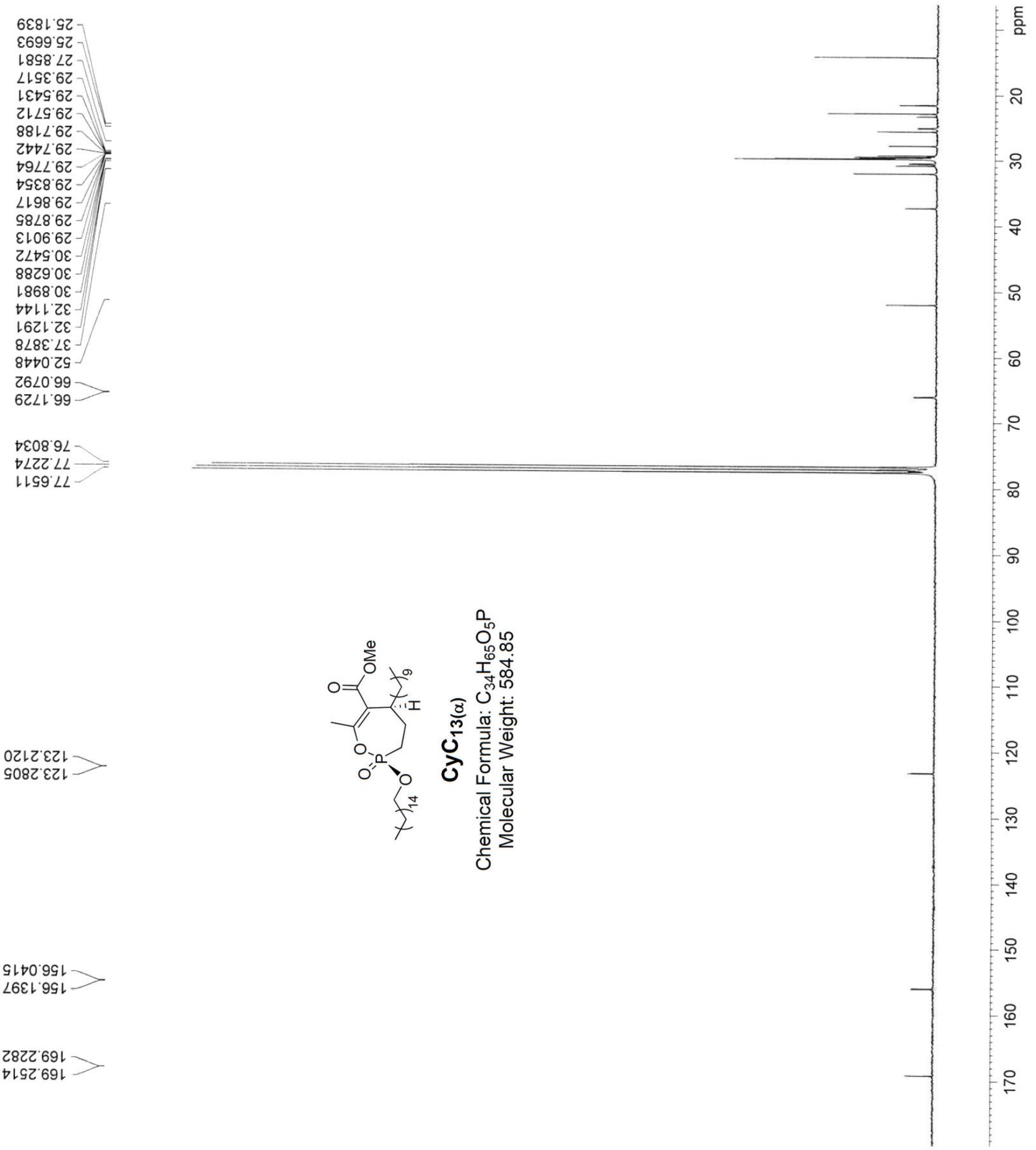

Figure S1B. ^{13}C NMR spectrum of **CyC$_{13(\alpha)}$** recorded at 75 MHz, and using $CDCl_3$ (δ = 77.23 ppm) as an internal standard of solvent.

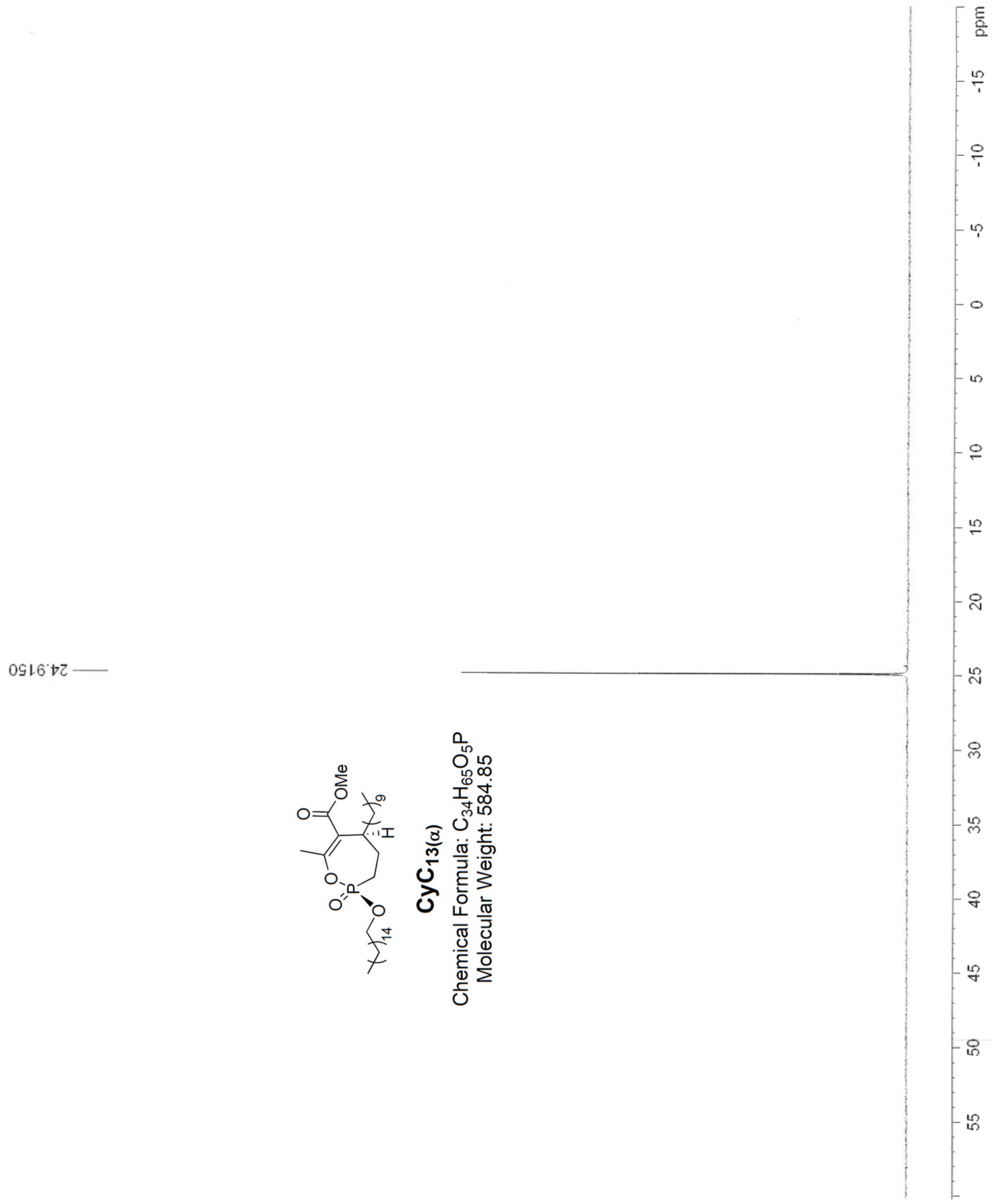

Figure S1C. ^{31}P NMR spectrum of **CyC$_{13(\alpha)}$** recorded at 121 MHz and referenced to external 85% H_3PO_4 (0 ppm).

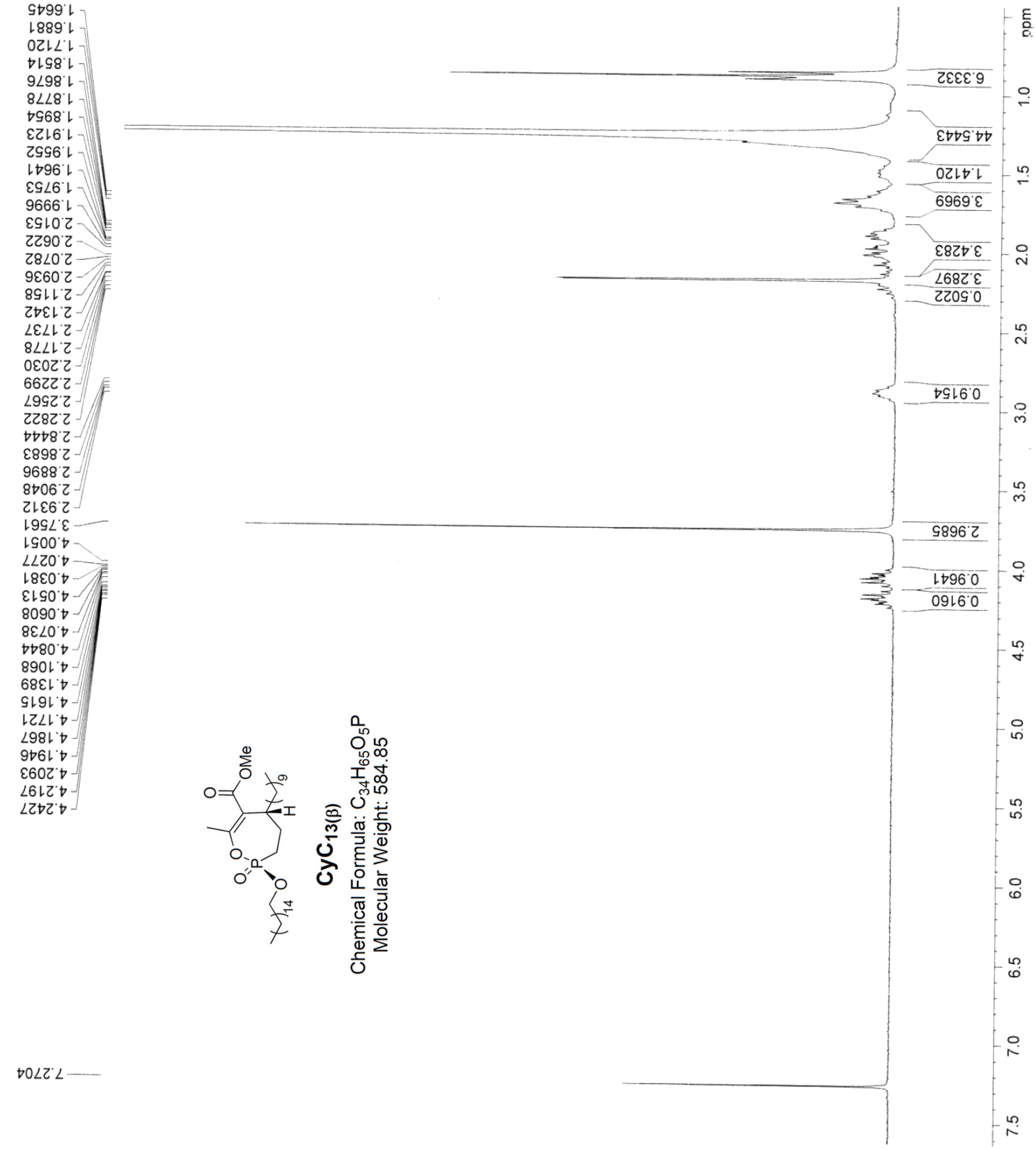

Figure S2A. ^{1}H NMR spectrum of **CyC**$_{13(\beta)}$ recorded at 300 MHz, and using $CDCl_3$ (δ = 7.27 ppm) as an internal standard of solvent.

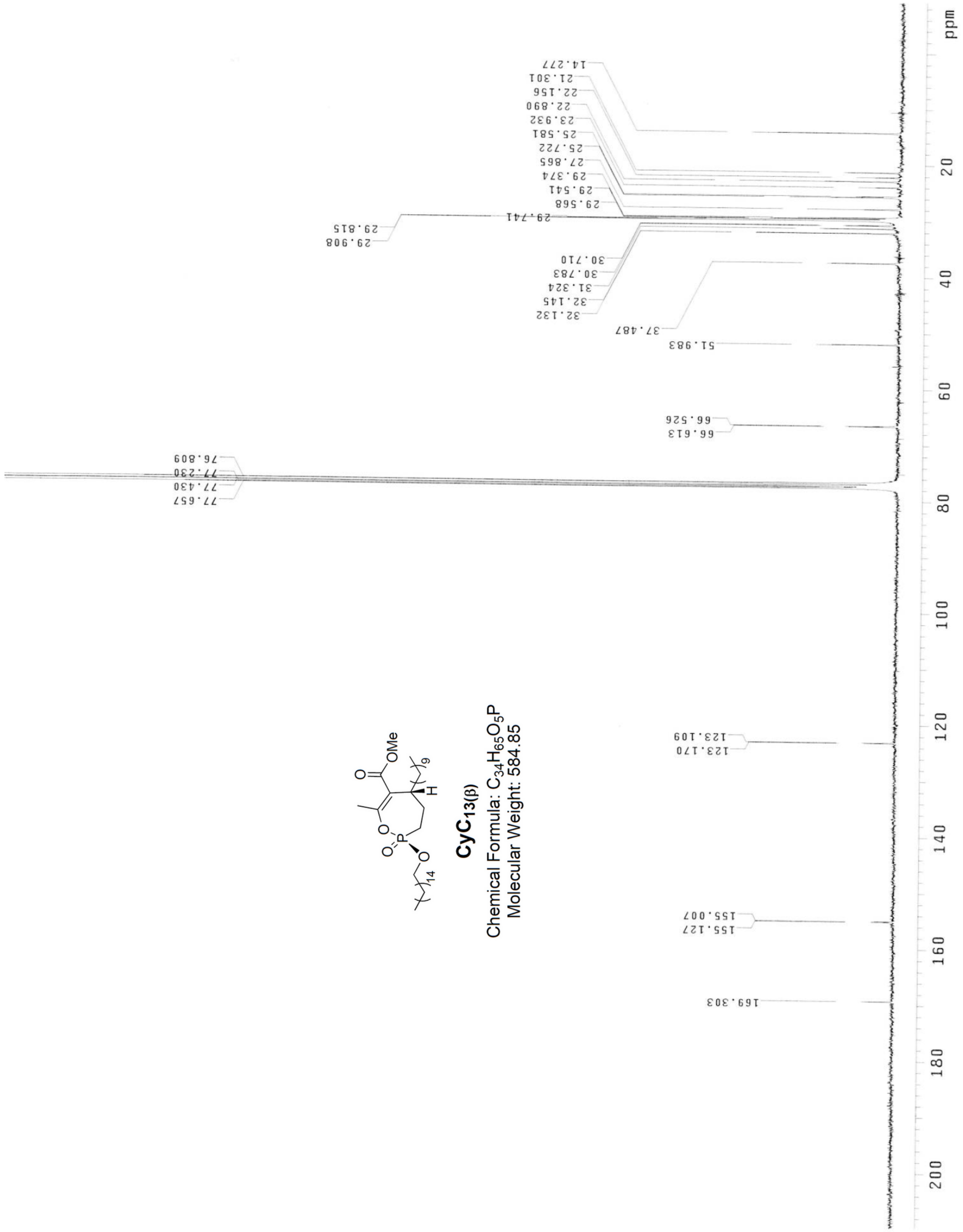

Figure S2B. ^{13}C NMR spectrum of **CyC$_{13(\beta)}$** recorded at 75 MHz, and using $CDCl_3$ (δ = 77.23 ppm) as an internal standard of solvent.

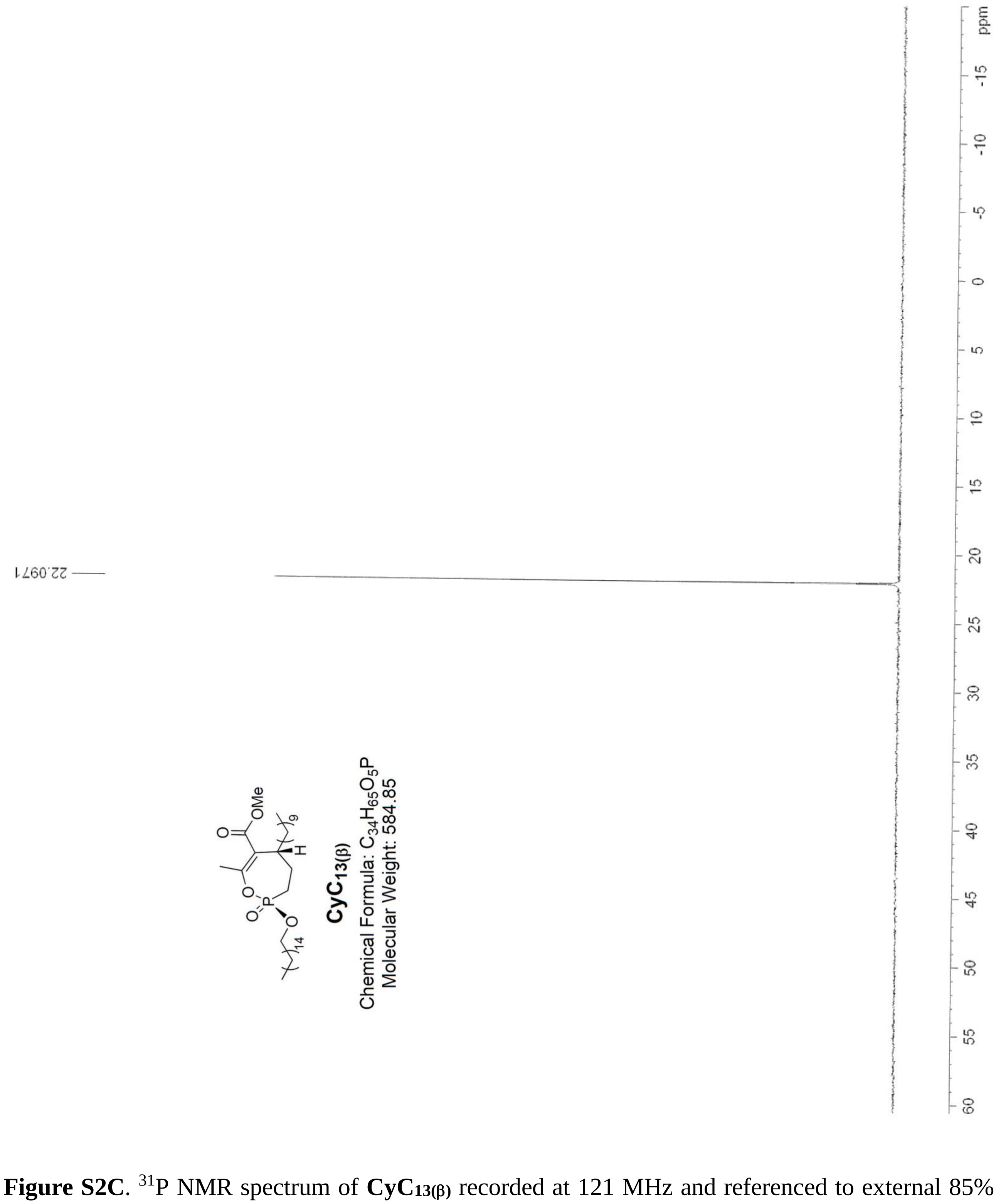

Figure S2C. ^{31}P NMR spectrum of **CyC$_{13(\beta)}$** recorded at 121 MHz and referenced to external 85% H_3PO_4 (0 ppm).

Table S2. Primers used in this study. Restriction sites if present are underlined.

Gene	Primer	Sequence (5′-3′)	Restriction site
Ag85A-F	Forward	5’-CCCAGCTTGTTGACAGGGTTCGTG -3’	
Ag85A-R	Reverse	5’-ACCATGGATCCCTAGGCGCCCTGGGGCGCG-3’	BamHI
Ag85C-F	Forward	5’-CCACGTTCTTCGAACAGGTGCGAAG-3’	
Ag85C-R	Reverse	5’-ACCATGGATCCTCAGGCGGCCGGCGCAGCAG-3’	BamHI
Rv0183-F	Forward	5’-GGAAATCATATGACTACCACCCGGACTG-3’	NdeI
Rv0183-R	Reverse	5’-CGGCGGGATCCCCGACAACCGCTCGGTGAGCC-3’	BamHI
LipH-F	Forward	5’-GGAAATCATATGACAGAGCCGACCGTCG-3’	NdeI
LipH-R	Reverse	5’-CGGCGGGATCCCTGATGCGTGCAACGCCCTCTTC-3’	BamHI
TesA-F	Forward	5'-CCAGCATATGCTGGCCCGTCACGGACCACG-3'	NdeI
TesA-R	Reverse	5'-CCAGAAGCTTAGCTCGATCATGCCATTGGAGTGTT-3'	HindIII
HsaD-F	Forward	5'-CCAGCATATGACAGCTACCGAGGAATTGACGT-3'	NdeI
HsaD-R	Reverse	5'-CCAGAAGCTTTCTGCCACCTCCCAGAAATTCAATC-3'	HindIII

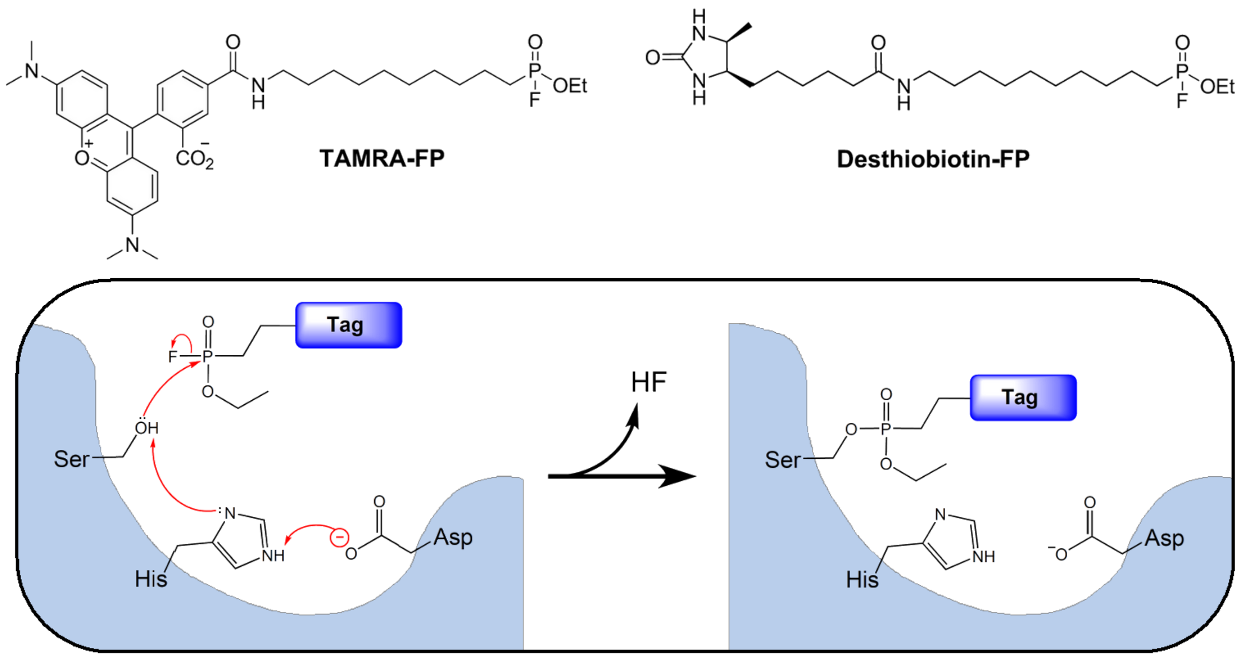

Figure S3. Chemical structures of TAMRA-FP for in-gel fluorescence detection and Desthiobiotin-FP for target enrichment. Each probe forms an irreversible covalent bond with an active site serine (or cysteine) for irreversible enzyme labelling.

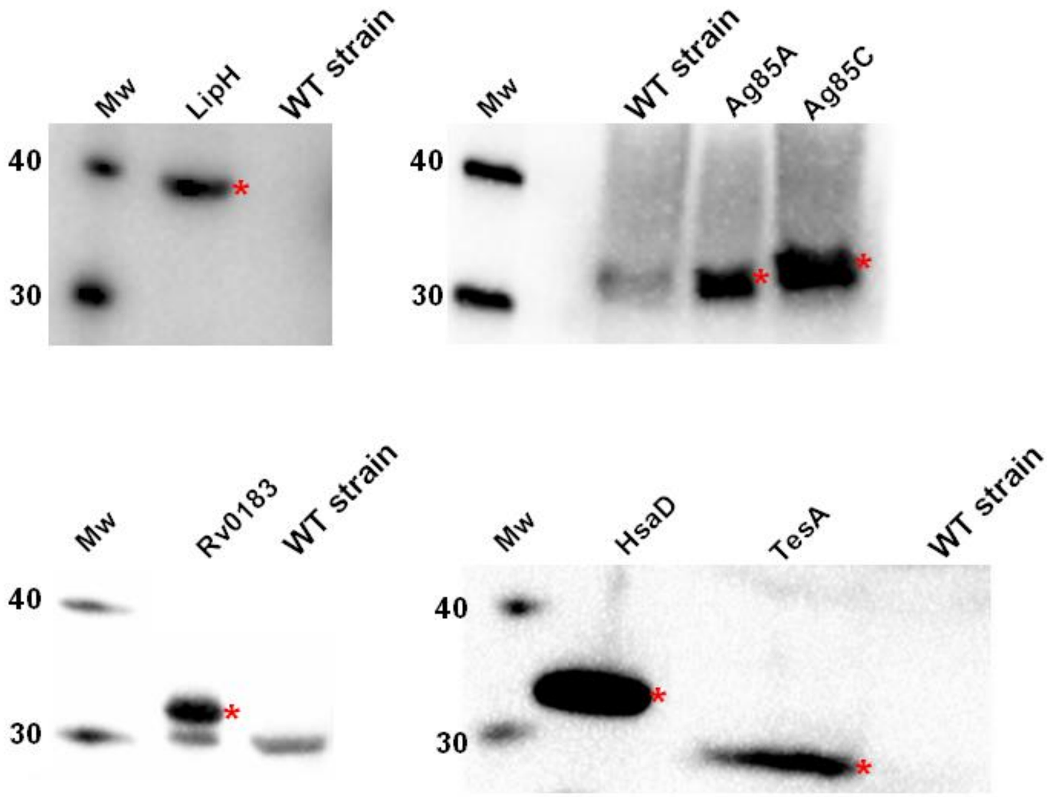

Figure S4. Western blot analysis of *M. tb* mc^{2}6230 overexpression strains as described in **Material and Methods** section. Each overexpressed protein is indicated with a red star and compared to the *M. tb* mc^{2}6230 wild type strain. Ag85A and Ag85C overexpression strains were revealed using specific monoclonal antibody directed against Ag85 complex; specific anti-Rv0183 rabbit polyclonal antibodies were used in the case of Rv0183 overexpression strain; while the other three overexpressed proteins (*i.e.*, LipH, TesA and HsaD) were revealed by HisProbe-HRP conjugated (Thermo-Fisher Scientific).
